# Integrated proteomic and targeted Next Generation Sequencing reveal relevant heterogeneity in lower-grade meningioma and ANXA3 as a new target in *NF2* mutated meningiomas

**DOI:** 10.1016/j.ebiom.2025.105814

**Published:** 2025-06-24

**Authors:** Maryam Shah, Yeasmin Akther, Claire L. Adams, Matthew C. Banton, Vikram Sharma, Emanuela Ercolano, David A. Hilton, Kathreena M. Kurian, Liyam Laraba, Waldemar Woznica, David Bryan Parkinson, Leandro José de Assis, Clemens Oliver Hanemann

**Affiliations:** a Brain Tumour Centre, Peninsula Medical School, University of Plymouth, Plymouth, United Kingdom; b School of Biomedical Sciences, University of Plymouth, United Kingdom; c Cellular and Anatomical Pathology, University Hospitals Plymouth NHS Trust, Derriford Road, Plymouth, PL6 8DH, United Kingdom; d Institute of Clinical Neuroscience, University of Bristol and Southmead Hospital, North Bristol Trust, Bristol, BS8 1QU, United Kingdom

**Keywords:** ANXA3, NF2, AKT1, KLF4, TRAF7, Meningioma

## Abstract

**Background:**

Meningiomas, the most common primary brain tumours, are classified by the World Health Organization (WHO) into grades 1, 2, and 3. Some grade 1 tumours exhibit increased clinical aggressiveness, with the biallelic mutation of *NF2* being the most frequently reported.

**Methods:**

In our study, we analysed the most common driver mutations (*NF2*, *AKT1*, *KLF4*, and *TRAF7*) in meningioma by genomics describing co-occurrences and new mutations. Furthermore, tumour tissue bearing the driver mutations was analysed by proteomics. The relevance of the specific target found in the most common driver mutation in meningiomas (*NF2*) was validated *in vitro* using both lower and higher-grade meningioma and further, the higher-grade meningioma was analysed *in vivo* using an NOD scid gamma (NSG) mouse model.

**Findings:**

Our genomic data revealed co-occurrences of non-*NF2* mutations in lower-grade meningiomas, suggesting synergistic effects supporting tumour growth. *NF2*^*−/−*^ meningiomas showed distinct proteomic clustering, with different mutations found in these clusters. Additionally, proteomics identified Annexin-3 (ANXA3) upregulated in *NF2*^*−/−*^ meningioma. Its role in proliferation was confirmed in grade 1 and subsequently grade 3 tumours *in vitro* and with abolished growth when knocked down in a meningioma mouse model.

**Interpretation:**

These findings highlight new targets in different meningioma backgrounds, presenting ANXA3 as a potential therapeutic target for meningioma treatment.

**Funding:**

This work was funded by the Brain Tumour Centre of Excellence.

**Research in context**

**Evidence before this study**

Meningioma is the most common primary brain tumour. Over the past seven years, several multi-omic analyses have been conducted on meningiomas, comparing different World Health Organization (WHO) grades and associated genetic subgroups, primarily focussing on methylation patterns and copy number variations (CNVs). These findings have been summarised in recent reviews. Biallelic mutations in the *NF2* gene are the most frequent cause of meningiomas across all grades. While low-grade meningiomas are the most prevalent type, they can exhibit clinical aggressiveness and progress to higher WHO grades.

**Added value of this study**

We add value by focussing on low-grade meningiomas and showing 1. Co-occurrences of mutations suggest synergistic effects that promote tumour growth. 2. Differently expression in genetically defined groups and proteomic clustering of *NF2*^*−/−*^ low-grade tumours with additional new mutations differentiating these clusters. 3. Role of ANXA3 in meningioma proliferation being a key factor in *NF2*^*−/−*^ meningiomas.

**Implications of all the available evidence**

Our work further molecular stratifies the most common meningioma WHO grade 1 and provides a therapeutic target for the most common mutation in meningioma i.e., *NF2* loss relevant for low grade as well as higher grades.

## Introduction

Meningiomas are the most common primary brain tumour derived from cells located at the layers of the brain composed of dura and arachnoid.^1^ Meningioma can be classified as grades 1, 2, and 3, according to the World Health Organization (WHO). However, some of the grade 1 meningiomas behave clinically aggressively and can also transform into higher and more aggressive WHO grades.^2^, ^3^, ^4^, ^5^ Likely, one of the reasons for this is the presence of somatic mutations, causing different clinical outcomes and providing therapeutical vulnerabilities for molecular therapy.^6^^,^^7^

Several studies reported the identification of molecular meningioma subgroups, showing the biallelic mutation of Neurofibromatosis 2 (*NF2*) as the most common genetic disturbance in meningiomas.^5^^,^^8^^,^^9^ Meningiomas without *NF2* mutations show other somatic driver mutations such as mutations on Tumour necrosis factor receptor-associated factor 7 (*TRAF7*), Krüppel-like factor 4 (*KLF4*^*K409Q*^), v-akt murine thymoma viral oncogene (*AKT1*^*E17K*^), and Phosphatidylinositol-4,5-bisphosphate 3-kinase catalytic subunit alpha (*PIK3CA*) which together represent 35% of driver somatic mutation burden in meningioma.^10^ The association of *AKT1*^*E17K*^, *KLF4*^*K409Q*^, and *PIK3CA* mutated genes have been observed to co-occur with *TRAF7* mutations, however, no co-occurrence has been observed between *AKT1*^*E17K*^ and *KLF4*^*K409Q*^.^10^^,^^11^ *NF2*, *AKT1*^*E17K*^, *KLF4*^*K409Q*^, and *TRAF7* are the most common mutations observed in meningiomas, and some other somatic driver mutations have been detected at low frequency, such as POLR2A, KDM6A, CHD2, PTEN, CREBPP, FBXW7, BAP1, FOXM1, and PIK3CA.^8^^,^^12^^,^^13^

PIK3CA is an upstream activator of the PI3K/AKT pathway and the *AKT1*^*E17K*^ mutation is responsible for the activation of downstream targets of this pathway, such as the mTOR pathway.^14^ *PIK3CA* and *AKT1*^*E17K*^ mutations are mutually exclusive,^10^ and although both mutations belong to the same pathway, methylation status shows that meningiomas with driver mutations in *PIK3CA* are reported as benign, while those with the *AKT1*^*E17K*^ mutation are classified as benign and intermediate^4^ reflecting different tumour aggressiveness.

Among *NF2*-deficient tumours, different histological subtypes have been found, indicating the presence of additional genetic features that might stratify *NF2*-deficient tumours into subgroups.^15^ The distribution of somatic mutations throughout the *NF2* gene shows that mutations along the *NF2* gene are correlated with clinical outcomes and classified as mild, moderate and severe in the severity group classification.^16^

A number of independent groups have integrated genomic, epigenomic, transcriptomic and proteomic data to define distinct molecular groups with unique biology and clinical outcomes through different WHO grades.^6^^,^^8^^,^^17^^,^^18^ Our study integrated proteomic, DNA methylation, and targeted Next Generation Sequencing data to analyse specifically lower-grade meningiomas to look for molecular heterogeneity in this defined group. We revealed specific signatures in lower-grade meningioma such as *NF2*, *AKT1*^*E17K*^, and *KLF4*^*K409Q*^, and identified co-occurring mutations such as ATM-BIVM-ERCC5, PIK3C2B-SDHD, and ERCC4-MET. We observed metabolic upregulation related to carbon metabolism in *AKT1*^*E17K*^ and more cellular process regulation related to *KLF4*^*K409Q*^ and *NF2* mutations. We found the presence of two subgroups within the grade 1 *NF2*-deficient tumours using proteomics and revealed a correlation to distinct genetic features.

Our proteomic analysis of *NF2*-deficient tumours revealed the upregulation of Annexin-3, impacting cell proliferation. These findings offer potential targets for personalised treatments in patients with meningioma.

## Methods

### DNA extraction

DNA was extracted from each tumour sample using DNeasy Blood & Tissue Kit (Qiagen) according to the manufacturer's instructions. The quantity and quality of extracted DNA were checked using a Nanodrop 2000 spectrophotometer (ThermoScientific, US).

### Endpoint genotyping

Endpoint genotyping Kompetitive Allele Specific PCR (KASP™) was performed on a Roche Life Sciences Light Cycler 480 II as previously described.^19^

### DNA sequencing and bioinformatic

A total of 118 meningioma samples were sequenced using the Illumina TruSight Oncology 500 panel, and raw sequencing data were analysed with the TruSight Oncology 500 v2.2 Local App. The Next Generation Sequence (NGS) data is available in the cBioportal (http://zadehportal2.ccbr.utoronto.ca/) User: Review Password: datareview1. Variant calling data were annotated using the Cancer Genome Interpreter (CGI) and ANNOVAR online tools.^20^, ^21^, ^22^ Genomic data underwent clinical enrichment analysis, with driver mutations filtered based on previously defined thresholds. The FATHMM-MKL score was used to distinguish between neutral and pathogenic mutations, as it demonstrates strong predictive capability according to studies by Dong et al. (2014) and Hassan et al. (2019).^23^^,^^24^ Variants with allele frequencies ≥0.05 were included to exclude false positives, and common germline mutations were filtered by setting a population allele frequency threshold of ≤0.05.^25^ Variants with allele frequencies >20% were classified as heterozygous, while those >80% were considered homozygous.^26^ Following filtration, 104 samples were selected for further analysis. Driver mutations were analysed using the maftools package in R (version 4.2.0) and compared with online databases COSMIC v.99 and cBioPortal to identify unique mutations.^27^^,^^28^ Significantly co-occurring mutations were detected using maftools' somatic interactions tool.^27^ The molecular functions of mutated genes were explored using Cytoscape's ClueGO v2.5.10.^29^

### Cell culture and sample selection (cell lines and meningioma tissue)

The study was performed on meningioma-immortalised cell lines and tumour-derived primary cells prepared accordingly as previously described.^30^^,^^31^ All cells were cultured in media supplemented with ZellShield antibiotic (Cambio, catalogue 13-0150) and were tested and confirmed to be mycoplasma-free using a mycoplasma detection kit (Lonza, catalogue LT07-118). Frozen normal meninges tissue was acquired from Analytical Biological Services Inc (Donor ID ABS60200003215). Ben-men-1 (BM1) *NF2*^*−/−*^ grade 1 meningioma (RRID: CVCL_1959),^32^ NCH93 *NF2*^*−/−*^ grade 3 meningioma (RRID: CVCL_E5ZE),^33^ CH157MN *NF2*^*−/−*^ grade 3 meningioma (RRID: CVCL_5723),^32^ and tumour-derived primary cells were cultured in DMEM media (Dulbecco's modified eagles' medium supplemented with foetal bovine serum-10%, Penicillin Streptomycin-1%) at 37 °C, atmospheric oxygen and 5% CO_2_. Stable cell lines bearing genome-integrated ANXA3 KD were established via lentivirus transfection selecting the cells with 4 ug/mL of puromycin (BM1) and 10 ug/mL of puromycin (NCH93 and CH157MN) for 3 days and the cell went through at least 5 passages under puromycin selection to guarantee stability of the genome-integrated system. The *LUC2* SCR and shANXA3 cells originate from the NCH93 and CH157MN wild type, which carries the *NF2* mutation. These cells underwent a two-step transfection process: first, they were modified to overexpress *LUC2* and gain blasticidin resistance. After several passages and selection, a second transfection introduced either GFP-SCR or GFP-shANXA3 with puromycin resistance. The final constructs, NCH93 *LUC2* GFP-SCR or GFP-shANXA3, were subjected to double selection with puromycin and blasticidin before *in vivo* experiments. The stock cells represent a pool of transfected cells with positive selection for puromycin and/or blasticidin.

### Orthotopic xenograft meningioma models

Mice were housed in specific pathogen-free conditions and fed a standard rodent diet with water *ad libitum*. Prior to xenograft transplantation, NCH93 *LUC2* cells (GFP-shSCR or GFP-shANXA3) were transduced with a plasmid to stably express luciferase (pLV[Exp]-Bsd-CMV > *Luc2*, VectorBuilder; VB900088-2625nuu) and selected using 10 ug/mL blasticidin (Gibco). Luciferase expression was verified via a reporter assay (Promega). A total of 1 × 10^6^ NCH93 cells in 5 mL of PBS were injected into the skull convexity of adult NSG mice (RRID: IMSR_JAX: 005557) using stereotaxic guidance as previously described.^34^ Mice were anaesthetised with isoflurane (4% induction, 2.5% maintenance) and received pre-operative analgesia (buprenorphine 0.05 mg/kg, meloxicam 5 mg/kg) and post-operative meloxicam 5 mg/kg. Daily checks were performed to monitor for adverse effects. Tumour growth was monitored using bioluminescence imaging (IVIS Spectrum Lumina III–Revvity) following intraperitoneal injection of d-Luciferin (150 mg/kg). Bioluminescence data were recorded as total flux and normalised to day 5 post-surgery readings. Tumours were harvested for analysis in RIPA buffer or 4% paraformaldehyde. The study included two arms: GFP-shSCR (n = 10) and GFP-shANXA3 (n = 8) xenografted mice. Mice were not randomised, but both sexes were equally represented. Researchers were aware of the group assignments. The primary outcome was the normalised bioluminescence fold-change in each group.

### Ethics

All animal experiments complied with UK Home Office regulations under the Animals (Scientific Procedures) Act of 1986 and were approved by the Plymouth University Animal Welfare and Ethical Review Board bearing the Home Office Project Licence number PP6780230 (Mechanisms of tumorigenesis in the nervous system). The study was conducted in compliance with ARRIVE guidelines.^35^ A total of 118 meningioma samples were collected for sequencing from Derriford Hospital in Plymouth and Southmead Hospital in Bristol. Samples were collected from patients with informed consent. All tumour samples were collected from either Plymouth Brain Tumour Bank (REC reference: 24/SC/0278; IRAS project ID: 345630) or Brain UK^36^ project reference 24/009 (REC reference: 24/SC/0044; IRAS project ID: 330020) in accordance with the Human Tissue Act (2004).

### Western blot

Tumour and cell lysates were prepared using RIPA lysis buffer (ref 89900, ThermoFisher) supplemented with a cocktail of protease & phosphatase inhibitors (ref 1861281, ThermoFisher) and quantified using BCA protein assay (Cat: 23227, ThermoFisher, US) according to the manufacturer's instructions. Samples for SDS-PAGE were carried out as described previously.^37^ Antibodies and dilutions used: anti-ANXA3 Rabbit 1:1000 (HPA013398 Merck), anti-Vinculin Mouse 1:800 (V9131 Merck), anti-GAPDH Mouse 1:10,000 (ab8245 Abcam), anti-Phospho-p44/42 MAPK 1:1000 (9101 Cell Signalling), anti-p44/42 MAPK 1:1000 (4695S Cell Signalling), anti-MCM2 1:1000 (3619 Cell Signalling), anti-CLIC3 1:1000 (HPA005963 Sigma), anti-CRABP2 1:1000 (HPA004135 Sigma), anti-GMDS 1:50 (NBP1-33424 Novus), anti-Pyruvate Carboxylase 1:500 (HPA043922 Sigma), anti-Endoglin 1:500 (HPA011862 Sigma), anti-E-cadherin 1:500 (SAB4503751 Sigma), anti-AEP2 1:500 (SAB2701184 Sigma), anti-Solute carrier family 29 1:500 (NBP3-02977 Novus), anti-Mouse HRP 1:5000 (#1706516 ThermoFisher), and anti-Rabbit 1:5000 (#1706515 ThermoFisher). All antibodies used in the study are commercially available and validated by the suppliers. A Simple Wes assay was conducted using a Wes instrument (ProteinSimple, San Jose, CA) following the manufacturer's protocol. The assay utilised the Simple Wes kit (SM-W004 SDS 12–230 KDa Wes Separation Module Kit) along with an 8°×°25 capillary cartridge, both procured from Bio-Techne. Detailed antibodies information is provided in Supplementary Table S1.

### Virus production and transfection

Lentivirus production was performed using lentiviral vectors transfected into 293FT cells. GIPZ plasmids, containing a CMV promoter driving GFP expression, a puromycin resistance marker, and small hairpin RNA (shRNA) sequences, were used. Constructs included a scramble control and ANXA3 shRNA variants: 622 (clone ID V2LHS_49622, sequence: ATAAGTTGGATAGTAGTTG), 695 (clone ID V2LHS_49695, sequence: TATGAGAAGAAGTAAGGTG), and 751 (clone ID V3LHS_331751, sequence: AATGCTTCTTGAACTCTGT) (Horizon Discovery). Plasmids were extracted from bacteria using the QIAprep Spin Miniprep Kit. 293FT cells (1.4 × 10^6^; passage < 15) were cultured in DMEM medium (10% FBS, no antibiotics) until 70% confluency in 10 cm Petri dishes. A transfection mix containing 26 μL packaging mix, 2.6 μg plasmid, and 16 μL Fugene 6 (Promega) in 182 μL optiMEM was prepared per dish, incubated for 15 min, and added to the cells. After 5 min of settling, the cells were incubated for 20 h. Media was replaced at 24 h (day 1) and 48 h (day 2) with DMEM supplemented with 20% FBS and penicillin/streptomycin. Viral particle-containing supernatants were collected at each time point, filtered, and stored at −80 °C. To infect target cells, 1.6 mL of viral particles was combined with 0.4 mL fresh medium and 16 μg/mL protamine sulphate. Cells were incubated with the virus for 48–72 h, and transfection was confirmed via GFP signal under fluorescence microscopy.

### EdU assay

Cell proliferation assay was performed using the Click-It plus Alexa Flour 594 Imaging kit (C10639 Thermo Scientific, UK) according to the manufacturer's instructions the cells were incubated with 10 mM EdU solution for 4 h 10,000 cells were seeded per well in a 24-well plate with 500 mL of media and grown for 24 h. Finally, the results were imaged using a fluorescent microscope and the images were quantified using ImageJ (https://imagej.net/ij/download.html). Experiments were carried out in biological replicates (at least n = 3) from 3 independent experiments.

### Mass spectrometry

Sample preparation and mass spectrometry for global proteomics was performed as described previously.^38^ In Parallel Reaction Monitoring (PRM) experiments, peptides were separated using the Vanquish nanoLC nano flow system (Thermo Scientific, UK). A 5 μL sample was prepared in 0.1% formic acid (FA) and 2% acetonitrile (in 0.1% FA) and loaded onto an Acclaim PepMap 100 μm × 2 cm, 3 μm C18 nano trap column at a flow rate of 5 μL/min, bypassing the analytical column. Peptide elution was performed using a trap column inline with an Acclaim PepMap C18 nano column (75 μm × 25 cm, 3 μm, 100 Å) under a linear gradient of 96% buffer A and 4% buffer B to 75% buffer A and 25% buffer B at 300 nl/min over 130 min. The sample was ionised in positive ion mode using an Easy-Spray ESI source (Thermo Fisher Scientific, UK) and analysed with an Orbitrap Fusion Lumos (Thermo Finnigan, Germany) operating under Xcalibur 4.5 software in PRM mode. MS spectra of intact peptides (*m*/*z* 350–1500) were acquired at a resolution of 120,000 with an automated gain control target of 1,000,000 ions. MS2 scans were performed in the Orbitrap at 30,000 resolution using HCD mode, with an isolation window of 1.8. Singly charged and unassigned ions were excluded. Conditions included a spray voltage of 2.3 kV, heated capillary temperature of 275 °C, and normalised HCD collision energy of 30%. A list of *m*/*z* values for PRM was generated using Data Independent Acquisition (DIA) on an independent sample cohort. A spectral library was created from the DIA dataset using Spectronaut (v19.4) and Spectrodive (v11, Biognosys AG).

### Proteomics analysis

A total of 27 samples were analysed by LC-MS-based label-free proteomics according to (Dunn et al., 2019; Sofela et al., 2021).^37^^,^^39^ The raw mass spectrometry data files were analysed using MaxQuant (version 1.6.2.10). Label-free quantification (LFQ) values were log2 transformed and imputed with normal distribution using the Perseus software suite (Version 1.6.2.3). Statistical significance of protein changes in abundance was calculated by a two-tailed t-test, with p-values adjusted for multiple testing by a Benjamini-Hochberg with a False Discovery Rate at 1%. Microsoft Excel was used to calculate log fold changes (FC) and log2 FC Th1 and log2 FC ≤ −1 with p-value < 0.05 were used as selection criteria for further downstream analysis. Entries with 0 for LFQ were kept and included in the fold change calculations. A total of 4162 proteins were identified among the sample groups of NMT, *NF2*^*−/−*^, *AKT1*^*E17K*^*/TRAF7*, and *KLF4*^*K409Q*^*/TRAF7*. 2909 proteins were identified within normal meninges (n = 2) whilst, in meningioma tissue, 3490 proteins were identified in *AKT1*^*E17K*^*/TRAF7* tumours (n = 7) and 3820 proteins were identified in *NF2*^*−/−*^ tumours (n = 11) and 3179 proteins were identified in *KLF4*^*K409Q*^*/TRAF7* tumours (n = 10). A total of 663 proteins statistically significant were identified by ANOVA between different meningioma genomic groups and NMT using Benjamini-Hochberg adjusted p-value < 0.05 then hierarchical clustering was performed using Perseus to identify 5 protein groups. A total of 13 samples were used for Parallel Reaction Monitoring (PRM) analysis for fast detection and a high degree of accuracy in predefined target peptide quantification. We generated an in-house spectral library and selected a minimum of 4 unique peptides (protein group-specific) corresponding to CD44, Endoglin and E-Cadherin 1. The mass spectrometry proteomics data have been deposited to the ProteomeXchange Consortium via the PRIDE^40^ partner repository with the dataset identifiers PXD058651 and PXD058653.

### Clustering NF2 and molecular functions categorisation

The NF2 clusters based on different proteomic expression profiles were checked for their genomics background using the TruSight Oncology 500 assay, as well as *NF2* gene screening by Central Manchester University Hospitals, NHS. The associated molecular functions with each cluster were investigated using DAVID (The Database for Annotation, Visualisation, and Integrated Discovery; https://davidbioinformatics.nih.gov/). Mutated genes from each cluster were submitted to DAVID followed by performing functional annotation analysis. From Gene Ontology (GO) result, the associated molecular functions were exported for downstream interpretation with p-value < 0.05. Finally, all the results were visualised using R software (version 4.2.0). The methylation profile of both NF2 clusters was identified by Illumina Human Methylation EPIC (University Hospital Heidelberg and clinical Cooperation Unit Neuropathology (G380), German Cancer Research Center (DKFZ), Im Neuenheimer Feld 224 69120 Heidelberg, Germany).

### Statistics

All the Statistical analyses for Western blots were performed using GraphPad Prism software (v.10). For non-normal distribution, a non-parametric test (Mann–Whitney) was utilised. Statistical significance was assessed using unpaired two-tailed t-tests for two-sample comparisons assuming normal distribution. One-way ANOVA with Dunnett's multiple comparisons test with simple pooled variance was employed for experiments with more than two samples assuming normal distribution, alongside non-parametric tests (Friedman's test) if data is not normal. Significance levels will be indicated by ∗ < 0.05, ∗∗ < 0.01, and ∗∗∗ < 0.001.

### Roles of funders

The funders supported the dissemination of the data to the non-scientific public and did not play any roles in study design, data collection, data analysis, interpretation or writing of report.

## Results

### Somatic driver mutations in meningioma

**Fig. 1:**
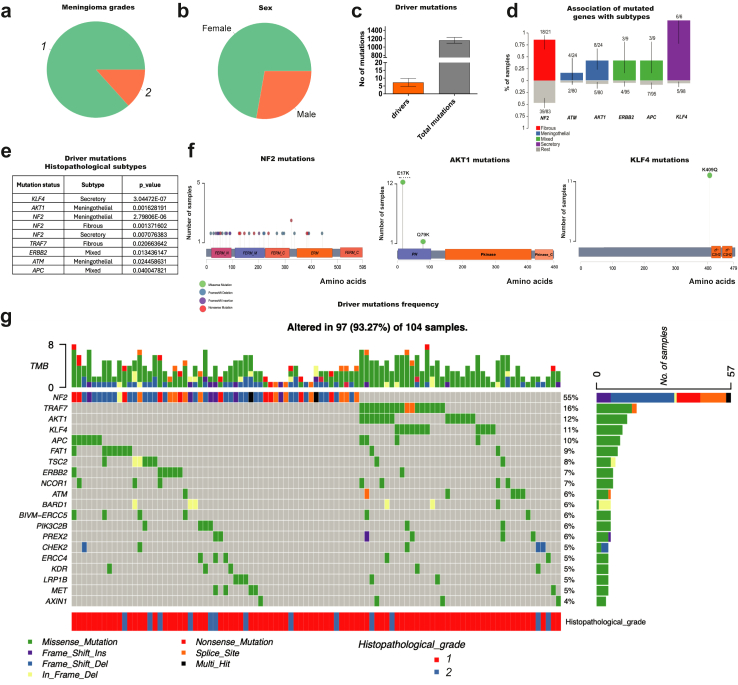
**Histopathological features and driver mutations frequency.** A total of 118 samples of meningioma tumours were collected, and samples were processed for DNA extraction and further analysis. **a)** Distribution of the population into meningioma grades, **b)** sex, **c)** number of driver mutations (0.58%) compared to the total mutations found in the population analysed. **d)** ClinicalEnrichment showing the classification of the histopathological subtypes based on the driver mutations. **e)** Histopathological subtypes for *AKT1*^*E17K*^, *KLF4*^*K409Q*^, *TRAF7*, and *NF2*^*−/−*^ mutations. **f)** Schematic diagram showing the distribution of mutations on the genes *NF2*, *AKT1*, and *KLF4*. **g)** Driver mutation frequency, histopathological subtypes, and the classification of the type of genetic modification found. TMB = tumour mutation burden. ClinicalEnrichment tool in maftools utilised Fisher's exact test with p-values adjusted for multiple testing by a Benjamini–Hochberg FDR at 1% to perform both pairwise and groupwise comparisons for d) and e), identifying association between genes and clinical subtype. Associations with a p-value < 0.05 was considered significant. The error bars represent the 95% confidence interval of binomial ratios while the bars are annotated with the number relative to the total number of samples.

After filtering, a total of 104 meningioma samples were analysed in this study. Details of the tumours are summarised (Fig. 1a–g) and more detailed information is provided (Supplementary Table S2). The driver mutations identified among total mutations represent 0.58%. Grade I samples showed the major driver mutations *NF2* (multiples sites of mutations), *AKT1* (two missense mutations E17K and Q79K), and *KLF4*^*K409Q*^ (single mutation K409Q) with previously described histopathological subtypes (Fig. 1c–e).

*NF2* mutations were mostly frameshift deletions and truncations, and most of the remaining driver mutations were missense mutations (Fig. 1g [green]). Driver mutations were associated with a specific tumour subtype presenting secretory subtype (*KLF4*^*K409Q*^) and meningothelial (*AKT1*^*E17K*^), however, multiple histological subtypes could be observed in the NF2-driver mutation group (Fig. 1d and e). The frequency of driver mutations observed in our cohort, as expected, shows that *NF2* (56%) is the most predominant, followed by *TRAF7* (16%), *AKT1* (12%), *KLF4* (11%), and *APC* (10%) (Fig. 1g). *NF2* driver mutations were checked for their associated severity score, and it was found most of the mutations belonged to severe category (score 3) (Supplementary Table S3).

**Fig. 2:**
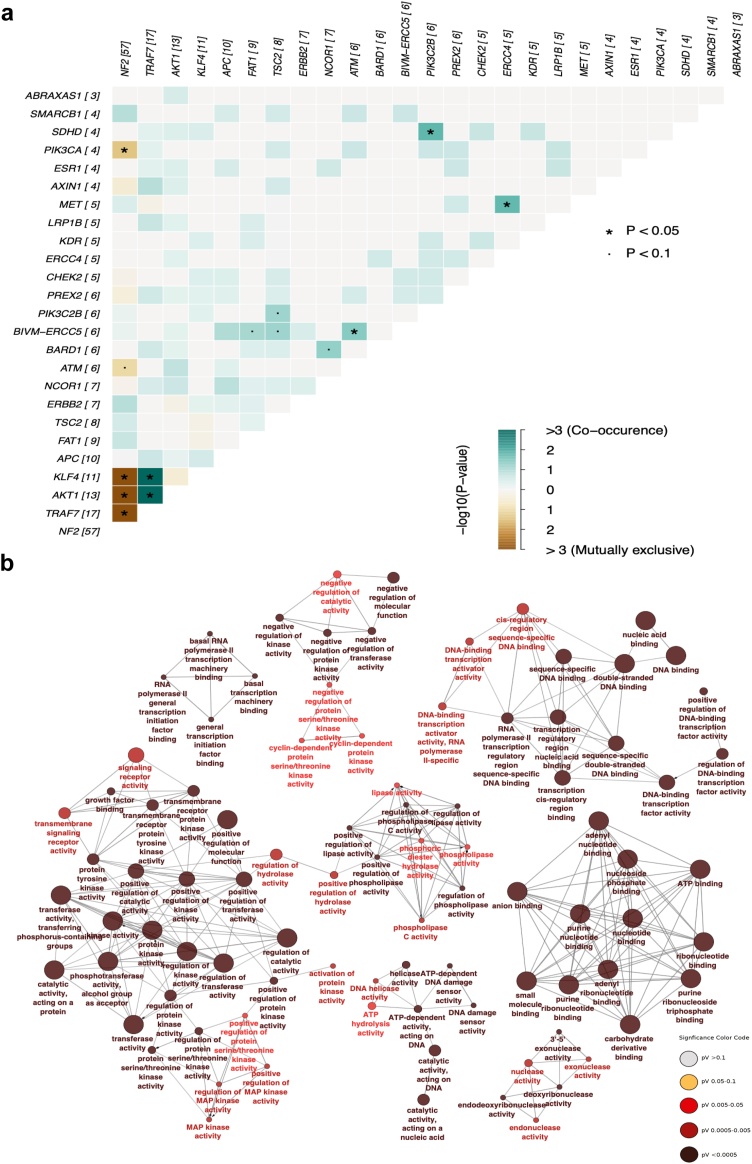
**Driver mutation co-occurrence and molecular functions. a)** Significantly mutually exclusive and co-occurring genes were identified using somatic interaction function in maftools (R package). The function applied pair-wise Fisher's Exact test with p-values adjusted for multiple testing by a Benjamini–Hochberg FDR at 1% to identify significant gene pairs and is represented as ∗p < 0.05 and ^·^p < 0.1 inside the squares. The numbers shown in brackets beside each gene indicate the number of samples in which a particular gene has been mutated. Green shows co-occurrence and brown is mutually exclusive. **b)** Further, gene enrichment analysis was conducted to explore the interactions between mutated driver genes and molecular functions using ClueGO v2.5.10, a Cytoscape tool. ClueGo employed a Two-sided hypergeometric statistical test to identify significant molecular functions enriched in dataset, with p-value cutoff 0.05 adjusted by Bonferroni step down method and Kappa Score Threshold 0.6. The node size represents the number of genes and the colour the level of significance.

We evaluated the correlation between these genetic alterations to look for synergistic effect via co-occurrence. As reported in previous publications, *NF2* driver mutations are mutually exclusive with the *PIK3CA*, *KLF4*, *AKT1*, and *TRAF7* genes.^41^ Furthermore, *TRAF7* mutation co-occurs with *KLF4* and *AKT1* as previously reported,^41^ showing the reliability of our analysis (Fig. 2a).

Among the co-occurrence pairs, we also found the presence of *ATM* and *BIVM-ERCC5* (Fig. 2a). ATM, a serine/threonine kinase participates in the DNA damage checkpoint controlling downstream signals responsible for entry into the S phase^42^ and *BIVM-ERCC5* gene encodes a protein involved in DNA repair and polymorphisms of this gene are related to an increased risk of some cancer types such as meningioma, glioma, neuroma, gastric, and breast cancers.^43^^,^^44^ Another co-occurrence observed in our dataset was *PIK3C2B* and succinate dehydrogenase *SDHD* (Fig. 2a). *PIK3C2B* encodes a phosphatidylinositol-4-phosphate 3-kinase catalytic subunit type 2 beta that belongs to the PIK3 pathway controlling downstream signalling from growth factor receptors such as EGFR and PDGFR.^45^ *SDHD* mutations have been associated with cancers, including neuroblastoma.^46^

Additionally, the co-occurrence of *ERCC4* and *MET* indicates the possible interplay between growth factor signalling and DNA repair (Fig. 2a). ERCC4 is also an endonuclease DNA repair, as is ERCC5 described above, and polymorphisms in both have been associated with tumours including meningiomas.^43^^,^^44^ MET is a receptor tyrosine kinase that interacts and recruits components of the PIK3/AKT pathway, leading to the activation of downstream targets on this pathway, this gene is also associated with growth factor receptors such as EGFR, regulating the activation of the MAPK pathway.^47^

The combination of mutated genes present in the different histopathological subtypes, in our cohort, represents a wide diversity of mutations distributed along the different histopathological subtypes identified (Supplementary Fig. S1). The molecular functions modulated by the driver mutations are distributed widely within categories which include regulation of kinase activity, control of DNA binding, transcription corepressor activity and nucleotide binding were among the most significant molecular functions. Importantly, all these pathways are involved in cell cycle regulation, proliferation, apoptosis, and DNA damage control (Fig. 2b).

### Proteomic analysis of driver mutations reveals subgroups

**Fig. 3:**
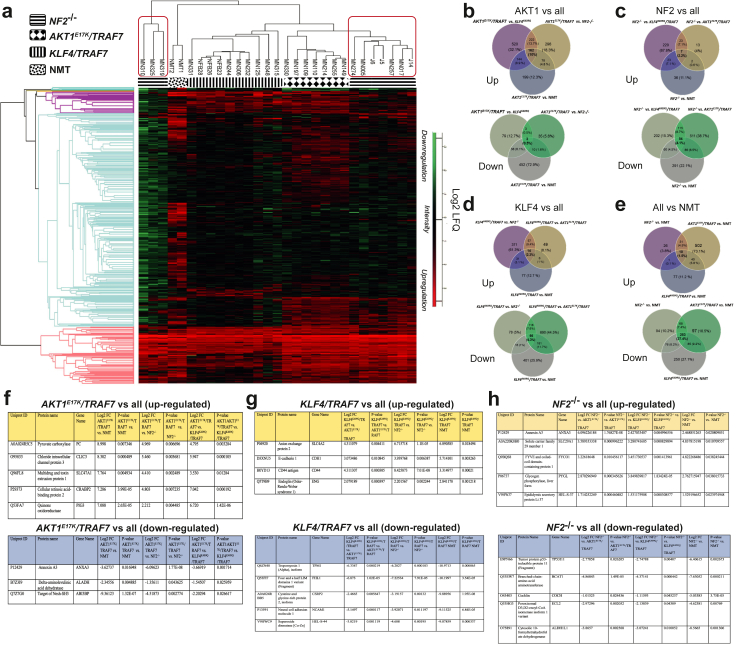
**Tissue proteomics from tumour-derived driver mutations.** Tumour tissue from samples holding *AKT1*^*E17K*^*/TRAF7*, *KLF4*^*K409Q*^*/TRAF7*, and *NF2*^*−/−*^ mutations were processed for mass spectrometry analysis and compared to normal meningeal tissue. **a)** Heatmap showing the differential expression profile for each meningioma background and the proteome clustering of the samples. Red squares show the *NF2*^*−/−*^ samples clustering into two subgroups. **b–e)** Venn diagrams representing each genetic background showing up and down-regulated proteins and their overlap in the middle (bold) with all data sets. **f–h)** Top 5 proteins up and down-regulated (if there are) for each genetic background overlapping with all data sets. Differentially expressed proteins for hierarchical clustering were obtained by submitting relative expression profiles to Perseus and performing an ANOVA (p-value < 0.05) on data imputation.

**Fig. 4:**
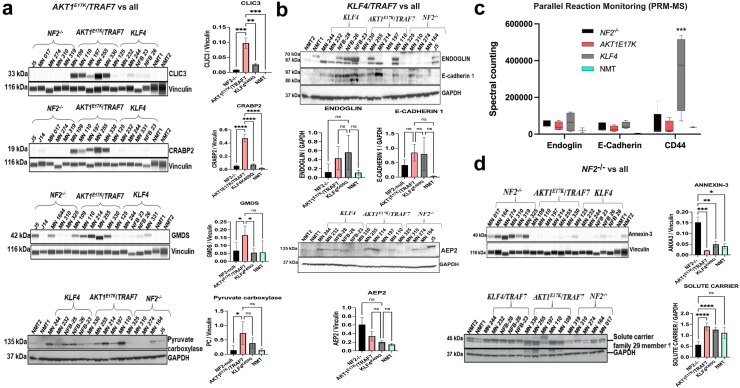
**Proteomic validation of up-regulated targets.** The same tumour tissue samples used for the proteomic assay were used for validation of the up-regulated targets by Western blot, Simple Wes, and PRM-MS. **a)** Tumours holding *AKT1*^*E17K*^*/TRAF7* genetic background (5 samples) were evaluated for four up-regulated targets: CLIC3, CRABP2, GMDS, and Pyruvate Carboxylase (PC) by Simple Wes and Western blot and **b)** Tumours with *KLF4*^*K409Q*^*/TRAF7* genetic background (5 samples) were used for validation by Western blot using three up-regulated targets: Endoglin, E-cadherin, and Anion exchange protein 2, **c)** Parallel Reaction Monitoring-Mass Spectrometry (PRM-MS) was carried out on an independent set of samples (5 *NF2*^−/−^, 4 *KLF4*^*K409Q*^*/TRAF7*, 4 *AKT1*^*E17K*^*/TRAF7*, and 2 NMT samples) for further validation of Endoglin, E-Cadherin, and CD44 expression and compared to all samples. **d)***NF2*^*−/−*^ tumours (5 *NF2*^−/−^ samples) were evaluated by Simple Wes and Western blot for two up-regulated targets: ANXA3 and Solute carrier family 29 member 1 blot. NMT = normal meningeal tissue, Endoglin and E-cadherin 1 were normalised by the same GAPDH. Experiments were carried out in three independent experiments. One-Way ANOVA with Dunnett's multiple comparisons test with simple pooled variance was employed for experiments with more than two samples assuming normal distribution, alongside non-parametric tests (Friedman's test) if data is not normal. Significance levels will be indicated by ∗ < 0.05, ∗∗ < 0.01, ∗∗∗ < 0.001, and ∗∗∗∗ < 0.0001.

A total of 4162 proteins were identified across 27 genetically defined meningiomas and two healthy normal meningeal tissue (NMT) used as controls, with a 1% false discovery rate (FDR), as determined by LC-MS-based label-free proteomics, as previously described.^37^ Tumour tissue samples carrying *AKT1*^*E17K*^*/TRAF7*, *KLF4*^*K409Q*^*/TRAF7*, or *NF2*^*−/−*^ mutations were processed for mass spectrometry analysis and compared to normal meningeal tissue (NMT). The proteomic analysis of these meningioma samples revealed different clusters associated with specific driver mutations (Fig. 3a). Notably, the *NF2* samples divided into two hierarchical groups with distinct protein profiles (Fig. 3a). Venn diagrams illustrate the overlap of up and down-regulated proteins detected by mass spectrometry (Fig. 3b–e). Multiple comparisons among tumours show that the tumour *AKT1*^*E17K*^*/TRAF7* shares more upregulated proteins (162 proteins) (Fig. 3b) and only 3 down-regulated proteins compared to other groups. The *KLF4*^*K409Q*^*/TRAF7* and *NF2*^*−/−*^ tumours exhibited more down-regulation of proteins (Fig. 3c and d). Moreover, comparing all tumours against normal meningeal tissue revealed 253 down-regulated proteins (Fig. 3e). These results showed upregulation in *AKT1*^*E17K*^*/TRAF7*, and transcriptional repression in *KLF4*^*K409Q*^*/TRAF7* and *NF2*^*−/−*^ tumours. Ingenuity Pathway Analysis (IPA) showed significant activation of the oxidative phosphorylation pathway in *AKT1*^*E17K*^*/TRAF7* tumours (Supplementary Fig. S2). *KLF4*^*K409Q*^*/TRAF7* tumours exhibited reduced activity score for several signalling pathways (Supplementary Fig. S3), while *NF2*^*−/−*^ tumours showed increase activity in pathways like Sirtuin signalling and Protein kinase A, albeit with generally lower protein level modulation (Supplementary Fig. S4). A comparison of the top five up and down-regulated proteins for each data set identified potential targets for drug intervention (Fig. 3f–h). These significantly up-regulated proteins were validated by Western blots, Simple Wes and Parallel Reaction Monitoring-Mass Spectrometry (PRM-MS).^48^ In tumours with *AKT1*^*E17K*^*/TRAF7* mutations, specific antibodies were available for four up-regulated proteins (CLIC3, CRABP2, GMDS, and Pyruvate carboxylase) (Fig. 4a). CLIC3, CRABP2, and GMDS were confirmed by Simple Wes (Fig. 4). Pyruvate carboxylase (PC) expression was significantly higher compared to *NF2*^*−/−*^ and a trend was observed for *KLF4*^*K409Q*^*/TRAF7* and NMT by Western blot analyses (Fig. 4a). For tumours with mutations *KLF4*^*K409Q*^*/TRAF7*, Endoglin, E-cadherin and Anion exchange protein 2 were selected for further validation by Western blot. All proteins showed heterogeneity among the samples when validated by Western blot, though trends against NMT were observed (Fig. 4b). We validated CD44 with higher expression in *KLF4*^*K409Q*^*/TRAF7* tumours by PRM-MS only due to limited sample availability and it displayed high significant upregulation compared to other targets tested (Fig. 4c). Interestingly, Anion Exchange Protein 2 was not detected using PRM-MS. Validation of upregulated proteins in *NF2*^*−/−*^ tumours revealed a markedly higher expression of Annexin-3 (ANXA3) compared to all datasets, but Solute carrier family 29 member 1 was not confirmed (Fig. 4d). In summary, we validated five highly expressed proteins as specific targets for different tumour mutational backgrounds: CLIC3, CRABP2, and GMDS for *AKT1*^*E17K*^*/TRAF7* tumours, CD44 for *KLF4*^*K409Q*^*/TRAF7* tumours and ANXA3 for *NF2*^*−/−*^ tumours. Given our focus on different WHO grade I tumours, *NF2* mutations being the most frequent and the proteomics data indicating two groups within *NF2*^*−/−*^ grade 1 meningioma, we decided to pursue further understanding of these two distinct *NF2*^*−/−*^ groups and continue validating the highly expressed specific target found up-regulated in *NF2*^*−/−*^ meningiomas, ANXA3.

### Grade 1 meningiomas with *NF2* mutations split into two subgroups with different mutations

**Fig. 5:**
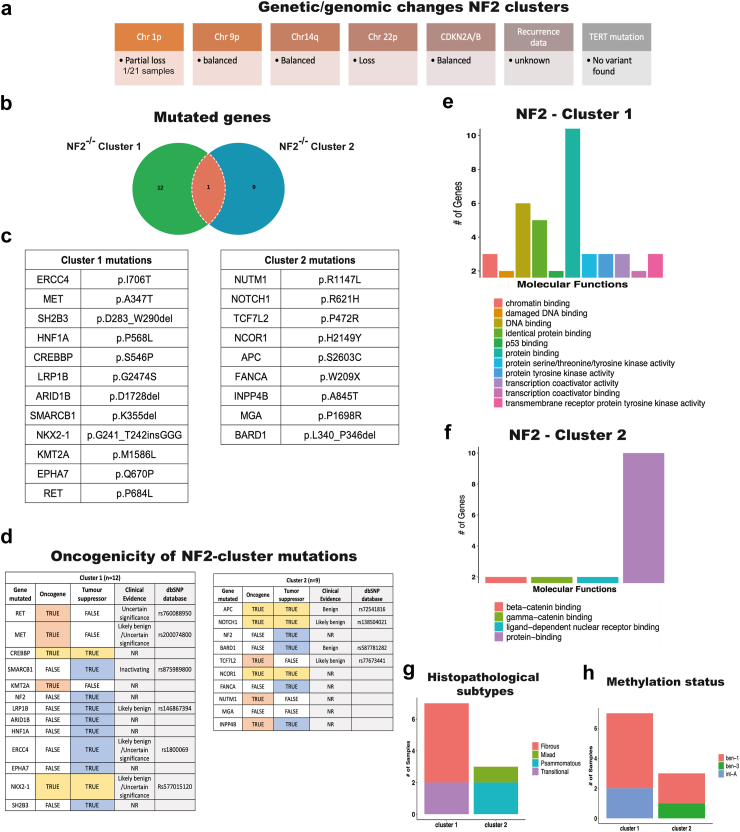
**NF2 splits into two subgroups.** The proteomic analysis and hierarchal characterisation generated two clusters for *NF2*^*−/−*^ samples, **a)** further genetic/genomic analysis was done on samples into these two groups. **b)** Ven diagram showing the number of mutated genes in NF2-clusters 1 and 2. **c)** Mutations presented by NF2-Cluster 1 (7 samples) and NF2-Cluster 2 (3 samples). **d)** Oncogenicity analysis of the NF2-clusters 1 and 2 showing mutated genes on oncogenes (orange), tumour suppressors (blue) and both (yellow). Clinical evidence is provided and the access number of the references from the dbSNP database (https://www.ncbi.nlm.nih.gov/snp/) is shown (NR = not reported). Genes found in both clusters were submitted to molecular function characterisation for specific molecular functions affected by these mutations: **e)** NF2-Cluster 1 and **f)** NF2-Cluster 2. The number of genes within each molecular function is shown. **g)** Histopathological subtype stratification and **h)** Methylation status for NF2-clusters.

*NF2* mutation severity score, loss of chromosome 1p, deletion of *CDKN2A/B*, and global methylation status have been identified as negative prognostic factors for meningioma.^2^^,^^16^ Additionally to these chromosomal abnormalities, *TERT* mutations and common Copy-Number Variation (CNV) are known to influence tumour aggressiveness^15^^,^^49^^,^^50^ and could have potentially explained the hierarchical cluster in our proteomic data (Fig. 3a) (Supplementary Table S4). In our analysis of genetic changes across *NF2* proteomics clusters, most samples retained an intact chr1p (one sample with partial loss), both clusters showed intact 9p and 14q, none displayed alterations in *CDKN2A* or *TERT* mutations (Fig. 5a). To explore other mutational features underlying clusters, we analysed mutations in samples from the two *NF2*^*−/−*^ tumour subgroups identified by hierarchical proteomic clustering (cluster 1 and cluster 2). Each cluster revealed distinct sets of driver mutations, with unique mutated genes beyond *NF2* itself (Fig. 5b and c). We subjected mutations in each cluster to oncogenicity analysis (oncoEnrichR 2023.06). NF2-cluster 1 showed at least one documented inactivating mutation in the tumour suppressor gene *SMARCB1* (Fig. 5d). The classification of molecular functions of the mutated genes in each cluster yielded different results. NF2-cluster 1 shows molecular functions related to at least 11 categories, including DNA damage, DNA binding, p53 binding, and transcription co-activation (Fig. 5e). In contrast, NF2-cluster 2 exhibited a more limited range, with only four categories: β-catenin binding, gamma-catenin binding, nuclear receptor binding, and protein binding (Fig. 5f). Histopathological subtype analysis of the NF2 clusters revealed fibrous and transitional subtypes in cluster 1 and psammomatous and mixed subtypes in cluster 2 (Fig. 5g). Methylation classification indicated that NF2-cluster 1 falls into the ben-1 and int-A categories, while NF2-cluster 2 is classified as ben-1 and ben-3 (Fig. 5h). These findings highlight the heterogeneity of *NF2* samples, indicating different mechanisms of tumorigenesis and demonstrating the complexity of *NF2* sample phenotypes.

### Targeting Annexin-3 in *NF2*^*−/−*^ tumour cells impairs proliferation

**Fig. 6:**
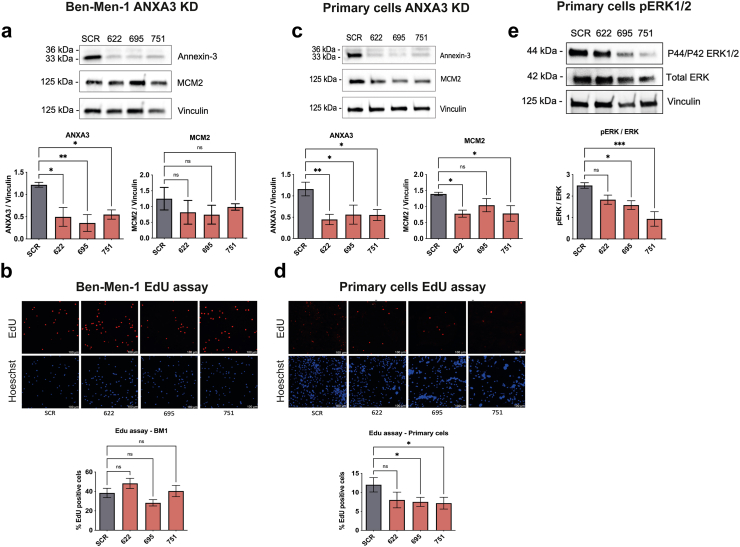
***NF2*-deficient tumour-derived cells require ANXA3 for proliferation.** ANXA3 KD constructions were designed into lentivirus and the efficiency of KD was tested in Ben-men-1 immortalised cell line. **a)** Ben-men-1 ANXA3 KD using three constructions 622, 695, and 751 the protein loading was normalised with vinculin for ANXA3 expression. The same constructions were tested for MCM2 expression and **b)** proliferation assay quantified by microscopy showing the % of Edu positive cells. **c)** ANXA3 KD in *NF2*^*−/−*^ tumour-derived primary meningioma cells with the quantification of ANXA3 normalised by vinculin and the evaluation of the proliferation marker MCM2. **d)** EdU proliferation assay showing the microscopy and % of EdU-positive cells. **e)** Inhibition of MAPK pathway via phosphorylation of ERK1/2 was evaluated in ANXA3 KD tumour-derived primary meningioma cells. Experiments were carried out in three independent experiments. One-way ANOVA with Dunnett's multiple comparisons test with simple pooled variance was employed for experiments with more than two samples assuming normal distribution, alongside non-parametric tests (Friedman's test) if data is not normal. Significance levels will be indicated by ∗ < 0.05, ∗∗ < 0.01, and ∗∗∗ < 0.001. Annexin expression measured as a double band at 36 and 33 KDa.

Proteomic analysis and validation of the *NF2*^*−/−*^ tumours revealed that Annexin-3 (ANXA3) is over-expressed in all tumours analysed compared to *AKT1*^*E17K*^*/TRAF7*, *KLF4*^*K409Q*^*/TRAF7* tumours, and normal meningeal tissue (Fig. 4d). ANXA3, also known as a member of the annexin family proteins referred to as lipocortin III and placental anticoagulant protein III, is involved in sustaining proliferative signalling through the activation of the Notch and MAPK/ERK/JNK pathways, contributing to tumorigenesis.^51^ This protein has been found up-regulated in a variety of cancers.^51^ To investigate the role of ANXA3 in low-grade *NF2*^*−/−*^ tumours, we designed genome-integrated lentivirus knockdown (KD) constructs. First, we created stable ANXA3 KD cell lines using Ben-men-1 (BM1) *NF2*^*−/−*^ grade 1 cells, with knockdowns observed after at least 5 passages post-transfection, indicating system stability (Fig. 6a). To evaluate the effect of ANXA3 KD on proliferation in BM1 cells, we performed Western blot to evaluate the expression of the proliferation marker MCM2^52^^,^^53^ and performed an EdU proliferation assay on the KD constructs compared to the control. The results from both MCM2 measurements by Western blot and EdU assays indicated a trend toward reduced proliferation in at least one of the tested constructs, though the decrease was not statistically significant (Fig. 6a and b). While ANXA3 KD was successfully achieved in this cell line, the BM1 immortalisation process involved *TERT* overexpression, which may have contributed to a bypass effect on the knockdown.^54^ Thus, extending this analysis to tumour-derived grade 1 primary meningioma cells, the same KD system was used, and proliferation was measured. ANXA3 KD was successfully achieved in tumour-derived primary cells, resulting in a reduction of the expression of the proliferation marker MCM2 (Fig. 6c) and significantly reducing proliferation as measured by the EdU assay (Fig. 6d). Furthermore, the phosphorylation of ERK1/2 was evaluated in tumour-derived primary cells with ANXA3 KD, showing reduction using at least two constructs compared to the controls (Fig. 6e). These results confirm the critical role of ANXA3 in the proliferation of *NF2*^*−/−*^ low-grade meningioma cells.

### ANXA3 KD *in vivo* reduces tumour growth in higher grades meningiomas

**Fig. 7:**
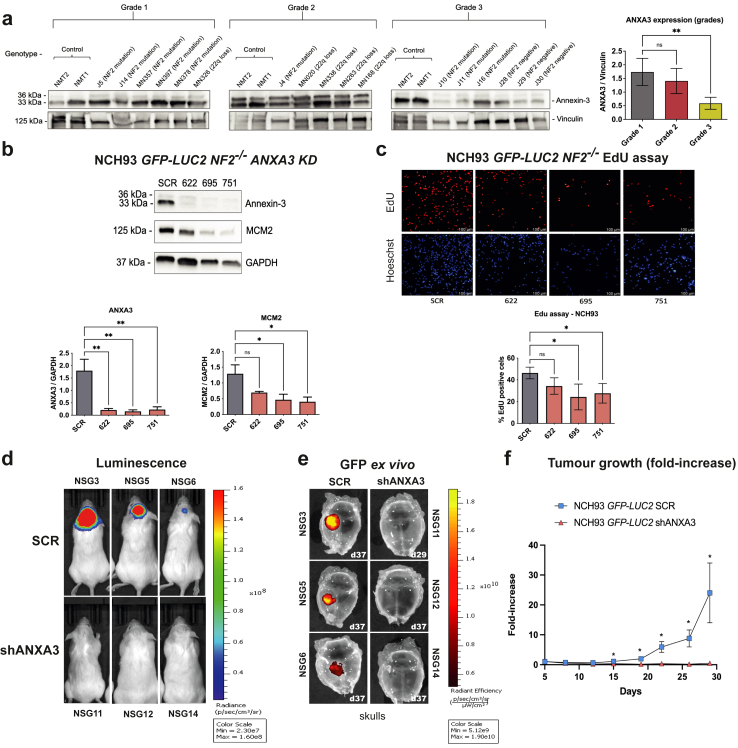
**ANXA3 KD *in vivo* reduces tumour growth in higher grades meningiomas. a)** Tumour tissue from meningioma grades 1, 2, and 3 was used for **a)** ANXA3 expression among grades followed by normalisation by vinculin. **b)** ANXA3 KD in NCH93 GFP-*LUC2 NF2*^*−/−*^ grade 3 immortalised cells over-expressing GFP with the quantification of ANXA3 and the evaluation of the proliferation marker MCM2 normalised by GAPDH in the same blot. **c)** EdU proliferation assay showing the microscopy and % of EdU-positive cells. Experiments were carried out in three independent experiments. **d)** Representative bioluminescence images of NSG mice engrafted with SCR (top panel) and ANXA3 KD (bottom panel) NCH93 *GFP-LUC2* cells, 29 days post-surgery, with luciferase signal directly correlating to tumour size. **e)** Representative fluorescent images overlaid on the skull convexity, showing the *ex vivo* GFP signal from tumour cells (days post-surgery indicated in the bottom right panel). **f)** Quantification of tumour growth represented as fold-increase in bioluminescence normalised to 5d post-surgery. A total of 10 animals with SCR cells and 8 animals with shANXA3 cells were used in 2 independent experiments and the cell growth was monitored up to 29 days post-surgery. Representative images of 3 animals showing the biggest (NSG3, 11), middle size (NSG5, 12), and smallest tumours (NSG6, 14) in the experiment for SCR and shANXA3, respectively. Scale bars show maximum and minimum luminescence and GFP signals, all images were set to the same scale. Statistical significance was assessed using unpaired two-tailed t-tests for two-sample comparisons assuming normal distribution. Significance levels will be indicated by ∗ < 0.05 and ∗∗ < 0.01. Annexin expression was measured as a double band at 36 and 33 KDa.

Although ANXA3 is highly expressed in grade 1 and 2 meningioma tissues as observed by Western blot analysis (Fig. 7a), the *NF2* phenotype, characterised by loss of function due to mutations, truncations, and/or deletions, is frequently observed in higher-grade meningiomas or more advanced molecular groups.^8^ To explore the potential relevance of targeting ANXA3 for higher-grade meningiomas, we extended our investigations to grade 3 meningioma cells. We created an ANXA3 KD stable cell line using NCH93 *LUC2 NF2*^*−/−*^ cells. We conducted proliferation experiments like those performed on primary cells, evaluating the proliferation marker MCM2 and using EdU assays. As with the primary cells, ANXA3 KD was successfully established in these cells, leading to significant reduction of MCM2 expression (Fig. 7b) and decreased proliferation as measured by the EdU assay (Fig. 7c). Among the ANXA3 KD constructs tested across the three cell lines (BM1, NCH93, and primary cells), construct 695 demonstrated the most pronounced phenotypic effects in NCH93 cells and was chosen for further *in vivo* validation using an NOD scid gamma (NSG) orthotopic xenograft mouse model. NCH93 *LUC2 NF2*^*−/−*^ cells engraft in NSG mice with ANXA3 KD showed reduced tumour growth after 29 days, as monitored by the IVIS imaging system compared to SCR controls (Fig. 7d). *Ex vivo* analysis, using GFP (which is a protein reporter over-expressed in our tumour cells), further confirming the reduction in tumour growth (Fig. 7e). Tumour fold-increase was measured starting from day 5 post-surgery (Supplementary Fig. S5), following cell engraftment in the animals (Fig. 7f). These findings confirm, both *in vitro* and *in vivo*, that ANXA3 is a viable target for controlling cell growth in higher-grade meningiomas.

## Discussion

Our Next Generation Sequence (NGS) panel detected all previously known meningioma driver mutations at the same frequencies as other studies (Fig. 1g), but we also identified some interesting driver mutations co-occurring with known tumour suppressor genes (Fig. 2a). A global proteomic analysis revealed unique protein expression patterns in all three genetically distinct meningioma groups *AKT1*^*E17K*^*/TRAF7*, *KLF4*^*K409Q*^*/TRAF7*, and *NF2*^*−/−*^ when compared to normal meningeal tissue allowing further exploration of these targets for personalised therapy (Fig. 3a).

Using proteomics, we showed that the *NF2*^*−/−*^ tumours divided into two distinct groups with distinct underlying mutational spectrum, NF2 clusters 1 and 2, independent of *NF2* severity score, chromosome loss, *CDKN2A/B* and *TERT* mutations (Fig. 5). Furthermore, this data identified Annexin-3 overexpressed in meningiomas with *NF2* mutations, which has been previously reported to be upregulated in other cancers and is known to enhance cellular proliferation through the Notch and MAPK/ERK/JNK pathways.^51^

In meningiomas with *AKT1*^*E17K*^*/TRAF7* mutations, we identified upregulation of CLIC3, an intracellular chloride channel involved in metalloprotease secretion, tumour migration, and invasion, as reported in ovarian, breast, and pancreatic cancers.^55^, ^56^, ^57^ Another overexpressed protein, CRABP2, from the retinoic acid family, is highly expressed in arachnoid and dura fibroblasts.^58^ Additionally, GMDS (GDP-mannose 4,6-dehydratase), a key enzyme in mannose metabolism, showed increased expression in these tumours. GMDS converts GDP-d-mannose to GDP-4-keto-6-deoxymannose and has been linked to higher-grade meningiomas through integrative transcriptomic and proteomic studies.^59^

Further analysis revealed upregulation of the oxidative phosphorylation pathway in *AKT1*^*E17K*^*/TRAF7* tumours compared to other genetic backgrounds and normal meningeal tissue (Supplementary Fig. S2). This suggests a higher metabolic profile, enhancing mitochondrial activity and enabling efficient utilisation of lipids and amino acids as carbon sources.^60^, ^61^, ^62^ These findings highlight critical targets and pathways requiring further validation and characterisation in meningioma tumorigenesis.

*KLF4*^*K409Q*^ tumours showed upregulation of CD44, a cell surface receptor interacting with matrix metallopeptidase 9 (MMP9) and positively correlated with proliferation, which is upregulated in higher-grade meningiomas.^63^ CD44 and MMP9 via degradation of the extracellular matrix could be involved in the peritumoral brain oedema where these patients showed higher expression of CD44.^64^

Meningioma growth is driven by the activation of multiple growth factor signalling pathways, leading to the stimulation of major proliferation pathways.^65^ We hypothesise that co-mutation patterns may play a role in growth factor signalling and/or DNA repair genes, which are known to be associated with the severity of the disease.^66^ These aspects remain understudied in meningioma. *BIVM-ERCC5-ATM* co-occurrence was observed and both genes are involved in DNA repair and mutations on these genes could amplify the number of genetic defects due to aberrant DNA repair. A potential collaboration between nutrient acquisition and activation of proliferative pathways such as PIK3K/AKT can be observed in the co-occurrence of *MET-ERCC4* (Fig. 2). Moreover, the co-occurrence of *PIK3C2B-SDHD* supports a link between growth factor signalling and metabolism (Fig. 2a).

Proteomic analysis revealed that *NF2*^*−/−*^ samples cluster into two subgroups based on protein expression (clusters 1 and 2) (Fig. 3a). Most *NF2* mutations in these subgroups were classified with a severity score of 3 (Supplementary Table 3). Molecular signature analysis highlighted differences between the clusters in oncogenicity, molecular functions, histopathological subtypes, and methylation status, suggesting greater aggressiveness for NF2-cluster 1 compared to cluster 2. Notably, NF2-cluster 1 included samples with inactivating *SMARCB1* mutations (Fig. 5d), which are associated with multiple meningiomas and atypical histopathological subtypes.^67^, ^68^, ^69^ However, clinical follow-up data for these patients is unavailable to confirm increased aggressiveness in NF2-cluster 1. The distinct clustering likely reflects the combined impact of mutations acting as pathway activators or inhibitors, resulting in the observed proteomic differences (Fig. 3a).

We identified different histopathological subtypes within NF2 clusters, with NF2-cluster 2 predominantly exhibiting the psammomatous subtype of meningioma. This subtype is characterised by the presence of psammoma bodies, which form calcified intracranial or spinal masses.^70^^,^^71^ This rare, benign type of meningioma is more frequently observed in women and predominant in our NF2-cluster 2 (Fig. 5g). In contrast, NF2-cluster 1 primarily shows a fibrous and transitional histopathological subtype. Combined with the methylation status, the samples in cluster 1 fall into the int-A and ben-1 classifications while cluster 2 fall into ben-1 and ben-3, suggesting a higher malignancy compared to NF2-cluster 2 (Fig. 5h). Thus, we believe co-occurring mutations other than chromosome 1p loss, *TERT* mutation, *CDKN2A/B* loss can stratify *NF2* mutated meningiomas, although a larger data set is required to confirm this hypothesis.

Knockdown of ANXA3, which is highly expressed in *NF2*^*−/−*^ tumours irrespective of specific *NF2* mutations (Fig. 4d, Supplementary Table S2 for clinical data), reduces proliferation as demonstrated by at least two proliferation assays (Fig. 6c and d). ANXA3 knockdown also decreases the expression of MCM2, a proliferation marker and early recurrence indicator,^52^^,^^53^^,^^72^ commonly used to identify higher molecular meningioma groups^8^ (Fig. 6, Fig. 7b). ANXA3 as previously described in other tumours^51^ and now confirmed in meningioma to control the activation of the ERK1/2 pathway (Fig. 6e), suggesting regulation of proliferation via this pathway resulting in increased DNA biosynthesis and the requirement of MCM2 protein for DNA replication (Fig. 6, Fig. 7b). This indicates that ANXA3 inhibition may serve as a promising therapeutic strategy for meningioma providing more specific treatment than conventional chemotherapy and this treatment could be used alongside the conventional treatments used such as radiation and surgical resection in higher grades.^73^ Although ANXA3 KD affects proliferation *in vitro* and *in vivo* using higher grade cells, it seems the function of ANXA3 is not related to its expression as ANXA3 levels are reduced in grade 3 tumour tissue (Fig. 7a). Comparison between NF2 clusters 1 and 2 also shows slightly significant reduction of ANXA3 in NF2-Cluster 1 in our mass spec data (Supplementary Fig. S8). Mechanistic characterisation of ANXA3 is required to understand specific downstream targets which could be achieved via usage of conditional ANXA3 knockout cells *in vivo* follow by omics analysis. Currently, a first-class ANXA3 inhibitor has been tested in triple-negative breast cancer, the small molecule SL18, reported as ANXA3-specific, showed low toxicity and efficiency *in vivo* by inactivating the Wnt/β-catenin pathway.^74^ The clinical application of the first-class ANXA3 inhibitor SL-18 has not been addressed in clinical trials to date, and the validation of an ANXA3-specific inhibitor for *in vivo* use with meningioma cells remains pending. Independent of genetic background or tumour grade, targeting ANXA3 could be a viable therapeutic approach for treating *NF2*^*−/−*^ tumours (Fig. 6, Fig. 7). The limitation of our study was the acquisition of tumour tissue with specific genetic backgrounds and the availability of clinical follow-up data of these samples. Our studies using tumour-derived primary cells highlight ANXA3 as an effective therapeutic target, and *in vivo* findings further support its potential, demonstrating significant effects on cell growth in higher-grade meningiomas (Fig. 7d–f). Emerging new therapies under investigation target cell proliferation via key regulators in proliferative tumour cells, however, implications of broad inhibition on healthy cells and the side effects are under study in clinical trials.^75^

In summary, we demonstrated the presence of the most common driver mutations in low-grade meningiomas, how these mutations can co-occur and their correlation with specific histopathological subtypes. The proteomic analysis provided valuable targets for further characterisation and investigation of their function in meningioma-tumour progression, additionally, showing the presence of a subgroup formation among *NF2*^*−/−*^ tumours. The protein ANXA3, regardless of heterogeneity was found to have increased expression in *NF2*^*−/−*^ tumours and showed promising results for personalised therapy driving attention to the development of a specific inhibitor that could support the treatment of patients with *NF2*^*−/−*^ tumours.

## Contributors

MS and YA: contributed equally to the execution of the experiments, data analysis, and manuscript writing. CLA, MCB, VS: these authors jointly supervised this work. EE, DAH, KMK; these authors contributed to clinical data, tumour tissue processing, and cell culture. LJA, LL, DBP, WW: these authors jointly supervised *in vivo* experiments. LJA: supervision and manuscript writing. COH: project design, supervision, funding, and manuscript writing. COH, LJA, CLA, and MS have accessed and verified the underlying data. All authors read and approved the final version of the manuscript.

## Data sharing statement

The data for this study are available upon reasonable request by contacting the corresponding author. Proteomics data can be accessed at ProteomeXchange Consortium via the PRIDE^40^ partner repository with the dataset identifiers PXD058651 and PXD058653. Next Generation Sequence (NGS) data is available in the cBioportal (http://zadehportal2.ccbr.utoronto.ca/) User: review Password: datareview1. Data sharing contact: oliver.hanemann@plymouth.ac.uk.

## Declaration of interests

The authors declare no competing interest. COH in 2022 received consulting fees from Recursion pharma. COH is member of the International Consortium of Meningioma. This is an academic consortium.

## Appendix A Supplementary data

Supplementary Fig. S1 **Categorisation of the mutated genes in each meningioma histopathological subtype.** The histology and location of meningiomas were used to classify the histopathological subtypes among the driver mutations observed in 104 samples.

Supplementary Fig. S2 **Proteomics IPA analysis *AKT1*^*E17K*^*/TRAF7*.** Ingenuity Pathway Analysis (IPA) using proteomic data from tumours holding *AKT*^*E17*^*/TRAF7* genomic background showing differential protein expression compared to *NF2*^*−/−*^, *KLF4*^*K409Q*^*/TRAF7*, and NMT. Balloon plot shows pathways demonstrating −log10 (p-value) > 3, Upregulated pathways (Z-score ≥ 3) marked by reddish to the yellowish dot; Downregulated pathways (Z-score ≤ −3) marked by purple to the blackish dot. NMT = normal meningeal tissue.

Supplementary Fig. S3 **Proteomics IPA analysis *KLF4*^*K409Q*^*/TRAF7*.** Ingenuity Pathway Analysis (IPA) using proteomic data from tumours holding *KLF4*^*K409Q*^*/TRAF7* genomic background showing differential protein expression compared to *NF2*^*−/−*^, *AKT1*^*E17K*^*/TRAF7*, and NMT. Balloon plot shows pathways demonstrating −log10 (p-value) > 3, Upregulated pathways (Z-score ≥ 3) marked by reddish to the yellowish dot; Downregulated pathways (Z-score ≤ −3) marked by purple to the blackish dot. NMT = normal meningeal tissue.

Supplementary Fig. S4 **Proteomics IPA analysis *NF2*^*−/−*^.** Ingenuity Pathway Analysis (IPA) using proteomic data from tumours holding *NF2*^*−/−*^ genomic background showing differential protein expression compared to *KLF4*^*K409Q*^*/TRAF7*, *AKT1*^*E17K*^*/TRAF7*, and NMT. Balloon plot shows pathways demonstrating −log10 (p-value) > 3, Upregulated pathways (Z-score ≥ 3) marked by reddish to the yellowish dot; Downregulated pathways (Z-score ≤ −3) marked by purple to the blackish dot. NMT = normal meningeal tissue.

Supplementary Fig. S5 **NSG Xenograft Mouse Model Controls. (a)** Animal weights (males and females) were monitored from the initial day (pre-surgery) to the final day, with representative animals from Fig. 7 shown in blue (SCR) and red (shANXA3). **(b)** Tumour growth was quantified through luciferase activity measurements using the IVIS imaging system 5 days post-surgery. **(c)** Representative animals from Fig. 7 imaged 5 days post-surgery using the IVIS system. A total of 10 animals with SCR cells and 8 animals with shANXA3 cells were used in 2 independent experiments. Scale bars show maximum and minimum luminescence signals; all images were set to the same scale. Statistical significance was assessed using unpaired two-tailed t-tests for two-sample comparisons assuming normal distribution. Significance levels will be indicated by ∗ < 0.05, ∗∗ < 0.01, and ∗∗∗ < 0.001.

Supplementary Fig. S6 **CH157MN *GFP-LUC2 NF2*^*−/−*^ ANXA3 KD *in vitro* shows reduced proliferation and *in vivo* loses ANXA3 KD. (a)** ANXA3 KD in CH157MN *GFP-LUC2 NF2*^*−/−*^ grade 3 immortalised cells over-expressing GFP with the quantification of ANXA3 and the evaluation of the proliferation marker MCM2 normalised by GAPDH. **(b)** EdU proliferation assay showing the microscopy and % of EdU-positive cells. Experiments were carried out in three independent experiments. One-Way ANOVA with Dunnett’s multiple comparisons test with simple pooled variance was employed for experiments with more than two samples assuming normal distribution, alongside non-parametric tests (Friedman's test) if data is not normal. **(c)** Representative bioluminescence images of NSG mice engrafted with SCR (top panel) and ANXA3 KD (bottom panel) CH157MN *GFP-LUC2* cells, 13 days post-surgery, with luciferase signal directly correlating to tumour size. **(d)** Representative fluorescent images overlaid on the skull convexity, showing the *ex vivo* GFP signal from tumour cells (days post-surgery indicated in the bottom right panel). **(e)** Quantification of tumour growth represented as fold-increase in bioluminescence normalised to 3d post-surgery and Western blots using samples from the same animals described in (d) showing the loss of ANXA3 KD *in vivo*. Total of 5 animals with SCR cells and 5 animals with shANXA3 cells were used in 1 independent experiment and the cell growth was monitored up to 13 days post-surgery and Representative images of 3 animals showing SCR and shANXA3, respectively. Scale bars show maximum and minimum luminescence and GFP signals, all images were set to the same scale. Statistical significance was assessed using unpaired two-tailed t-tests for two-sample comparisons assuming normal distribution. Significance levels will be indicated by ∗ < 0.05, ∗∗ < 0.01, and ∗∗∗ < 0.001. Annexin expression was measured as a double band at 36 and 33 KDa.

Supplementary Fig. S7 **NSG Xenograft Mouse Model Controls. (a)** Animal weights (males and females) were monitored from the initial day (pre-surgery) to the final day, with representative animals from Supplementary Fig. S6. **(b)** Tumour growth was quantified through luciferase activity measurements using the IVIS imaging system 3 days post-surgery. **(c)** Representative animals from Supplementary Fig. S6 imaged 3 days post-surgery using the IVIS system. Scale bars show maximum and minimum luminescence signals; all images were set to the same scale. Statistical significance was assessed using unpaired two-tailed t-tests for two-sample comparisons assuming normal distribution. Significance levels will be indicated by ∗ < 0.05, ∗∗ < 0.01, and ∗∗∗ < 0.001.

Supplementary Fig. S8 **Expression ANXA3 in the NF2 Clusters.** Mass spectrometry data analysis shown in Fig. 3 was used to quantify ANXA3 levels in NF2 Cluster 1 (7 samples) and NF2 Cluster 2 (3 samples). Statistical comparisons between the two clusters were performed using unpaired two-tailed t-tests, assuming a normal distribution. Statistical significance is indicated as follows: ∗p < 0.05, ∗∗p < 0.01, and ∗∗∗p < 0.001.

Supplementary Table S1 Antibodies validation list and animal description. The table contains detailed information on the antibodies and animals used in the project.

Supplementary Table S2 Samples from patients with meningioma and clinical features. Clinical and histopathological data for samples from patients used in this study. Genomic data (104 samples), mass spectrometry (27 samples), Parallel Reaction Monitoring (PRM) (14 samples), and ANXA3 expression in tumour tissue (17 samples).

Supplementary Table S3 Severity scores for *NF2*^*−/−*^ mutations identified in this study. The severity score for *NF2* mutations were found according to the Halliday et al, 2017.^16^

Supplementary Table S4 Samples from patients with meningioma and clinical features of NF2-clusters. Clinical, histopathological and oncogenicity data from patients with meningioma used for NF2-clusters. All samples are meningioma grade 1 *NF2*^*−/−*^. NF2-Cluster 1 (7 samples) and NF2-Cluster 2 (3 samples).

Supplementary Western blots

Reagent validation file
