# Supplementary material for: Integrated proteomic and targeted Next Generation Sequencing reveal relevant heterogeneity in lower-grade meningioma and ANXA3 as a new target in NF2 mutated meningiomas
Source: eBioMedicine. 2025 Jun 24;117:105814. doi: 10.1016/j.ebiom.2025.105814 (PMC12278414; doi:10.1016/j.ebiom.2025.105814)
Supplement: Supplementary Fig. S1 — Categorisation of the mutated genes in each meningioma histopathological subtype. The histology and location of meningiomas were used to classify the histopathological subtypes among the driver mutations observed in 104 samples. [file mmc1.pdf]

Subtype	Mutated genes
Atypical	PIK3C2B, NF2, CHEK2, KDR, FAT1, TP53, CDC73, ARID5B, ERCC4, TSC2, CDK12, EGFR, CTCF, ABRAXAS1, CREBBP, AKT1, FOXA1, TRAF7, MET, PREX2, ID3, FGFR3, FLT1, NCOR1, ERBB2, MLH1, RNF43, LRP1B, FANCD2, DHX15, ESR1, CBL
Fibrous	FGFR2, MET, NOTCH1, PTCH1, NF2, AR, CIC, APC, ROS1, LOC110117498-PIK3R3;PIK3R3, SMARCB1, NSD1, MSH2, ESR1, PREX2, TSC2, NCOR1, BARD1, MYC, PIK3CA, ERBB2, STK11, FANCC, POLE, BIVM-ERCC5;ERCC5, FAT1, NOTCH4, PTPN11, KDR, ERBB3, CDH1, HIST1H3B, TAF1, KDM5C, ERCC4, SH2B3, HNF1A, CREBBP, LRP1B, NKX2-1, KMT2A, INPP4A, GLI1, CEBPA, AXIN1
Meningiothelial	ATM, AKT1, PBRM1, KDM6A, NCOR1, ARID5B, TRAF7, BRIP1, EP300, SMARCA4, APC, KLF4, TET2, DDR2, MRE11, SPEN, FAT1, KMT2A, WT1, NF2, MET, EGFR, SMO, EPHA7, IDH2, PDGFRA, PALB2, SETBP1, BIVM-ERCC5;ERCC5, PREX2, TSC2, MSH2, PIK3CA, NKX2-1, PIK3C2B, ABRAXAS1, CHEK2, POLE, ERBB2, SOX17, AXIN1, MUTYH, NOTCH4, ERCC4, BARD1, CDKN2B, FOXA1, SETD2, FLT1, QKI, ABL1, DNMT3A, ESR1, BCORL1, ZFHX3
Psammomatous	GLI1, BIVM-ERCC5;ERCC5, DIS3, TSC2, POLD1, CHEK2, NF2, LRP1B, PIK3CA, NOTCH4, BCORL1, TRAF7, PTPRT, EP300, BARD1, APC, ABRAXAS1, SDHD, AKT1, ERCC3, ERCC2, NUTM1, NOTCH1, TCF7L2, MGA, PBRM1, FAT1, ROS1
Transitional	ATM, PIK3CA, SMARCA4, ROS1, ABL1, NF2, FAT1, ERCC4, PIK3C2B, AKT1, FOXA1, TET2, SDHD, EGFR, FGFR1, RAD50, KDR, MAP3K4, DIS3, AXIN1, TRAF7, CUX1, ERCC2, CBL, LRP1B, SF3B1, ARID1B, SMARCB1, ASXL1, EPHA7, RET, BAP1, POLE, TP53, POLD1, PTPRT, KLF4, MSH6, NTRK2, BIVM-ERCC5;ERCC5, SPEN, FANCD2, RASA1, NCOR1, PREX2, DNMT3A, BARD1, EZH2
Mixed	BARD1, NF2, TSC2, PIK3C2B, FANCC, AKT1, NCOR1, APC, ESR1, NOTCH1, TRAF7, SDHD, PALB2, RAD51C, KDR, BRCA2, ERBB2, VHL, TCF7L2, FANCA, INPP4B, SOX17, CSF3R, HNF1A, LZTR1, KLF4
Metaplastic	SDHD, STAT5B, PIK3C2B, CHEK2, MLH1, TRAF7, KLF4
Secretory	PMS2, TRAF7, KLF4, PREX2, JAK2, ERBB4, FBXW7, KDR, FGFR4, CHD2, FAT1, POLD1, PDGFRA, GLI1
Spinal	NF2
Parasaggital	NTRK1, FGFR2, KMT2A, NF2, NOTCH2, BRIP1, PTPRT
