# Supplementary figures and images for: Integrated proteomic and targeted Next Generation Sequencing reveal relevant heterogeneity in lower-grade meningioma and ANXA3 as a new target in NF2 mutated meningiomas
Source: eBioMedicine. 2025 Jun 24;117:105814. doi: 10.1016/j.ebiom.2025.105814 (PMC12278414; doi:10.1016/j.ebiom.2025.105814)

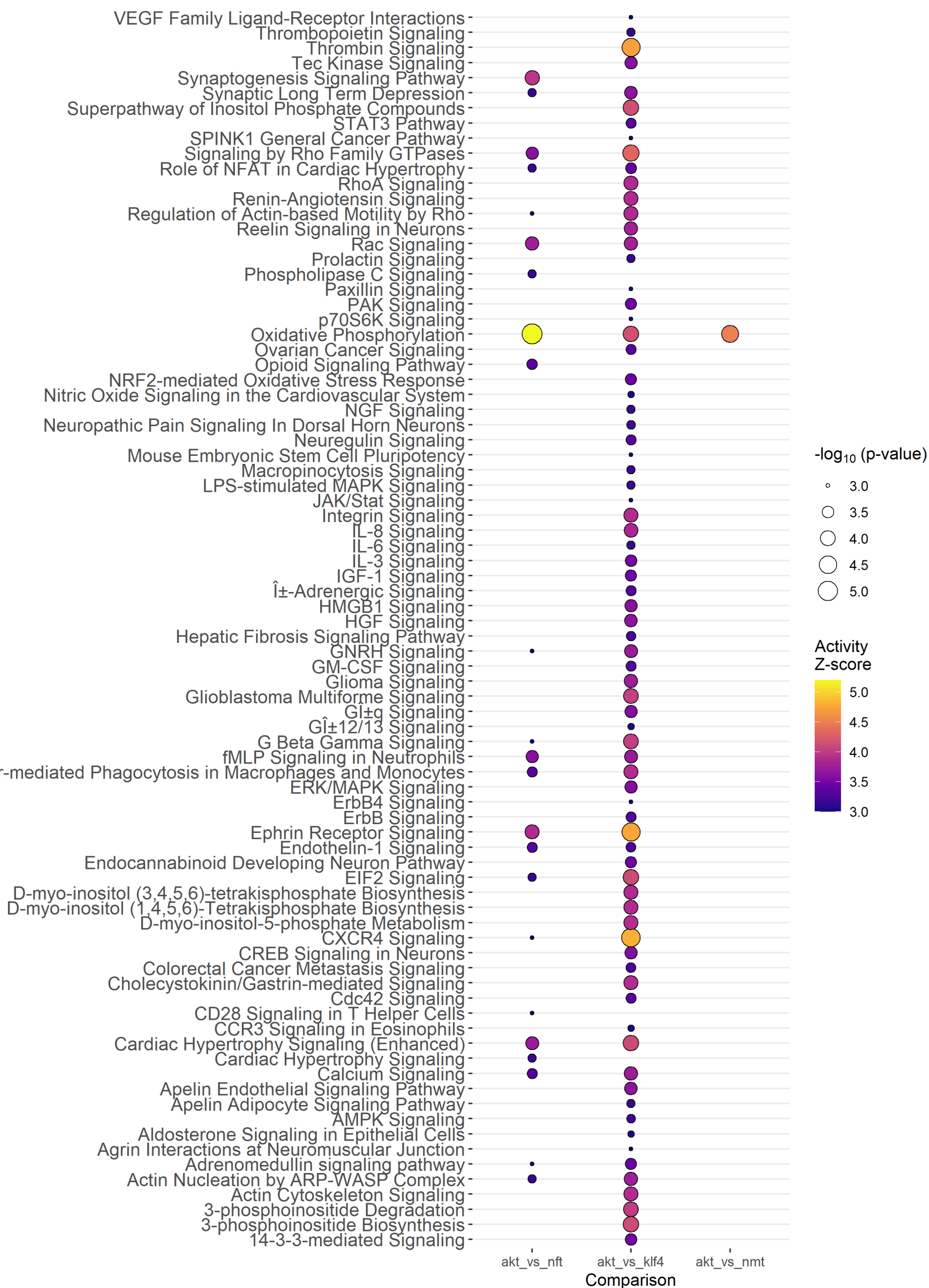

Supplement: Supplementary Fig. S2 — Proteomics IPA analysis AKT1E17K/TRAF7. Ingenuity Pathway Analysis (IPA) using proteomic data from tumours holding AKTE17/TRAF7 genomic background showing differential protein expression compared to NF2−/−, KLF4K409Q/TRAF7, and NMT. Balloon plot shows pathways demonstrating −log10 (p-value) > 3, Upregulated pathways (Z-score ≥ 3) marked by reddish to the yellowish dot; Downregulated pathways (Z-score ≤ −3) marked by purple to the blackish dot. NMT = normal meningeal tissue. [file mmc2.pdf]

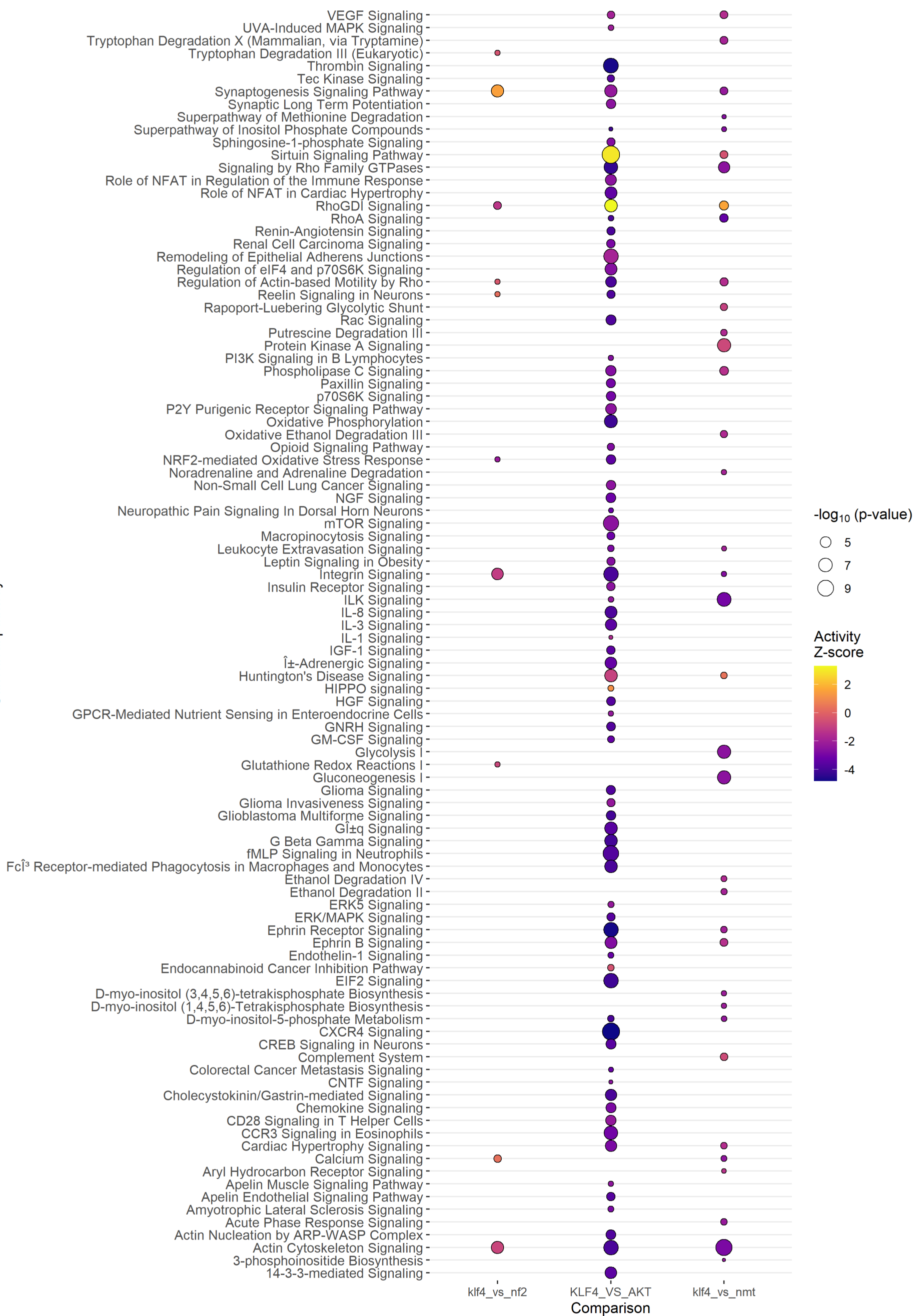

Supplement: Supplementary Fig. S3 — Proteomics IPA analysis KLF4K409Q/TRAF7. Ingenuity Pathway Analysis (IPA) using proteomic data from tumours holding KLF4K409Q/TRAF7 genomic background showing differential protein expression compared to NF2−/−, AKT1E17K/TRAF7, and NMT. Balloon plot shows pathways demonstrating −log10 (p-value) > 3, Upregulated pathways (Z-score ≥ 3) marked by reddish to the yellowish dot; Downregulated pathways (Z-score ≤ −3) marked by purple to the blackish dot. NMT = normal meningeal tissue. [file mmc3.pdf]

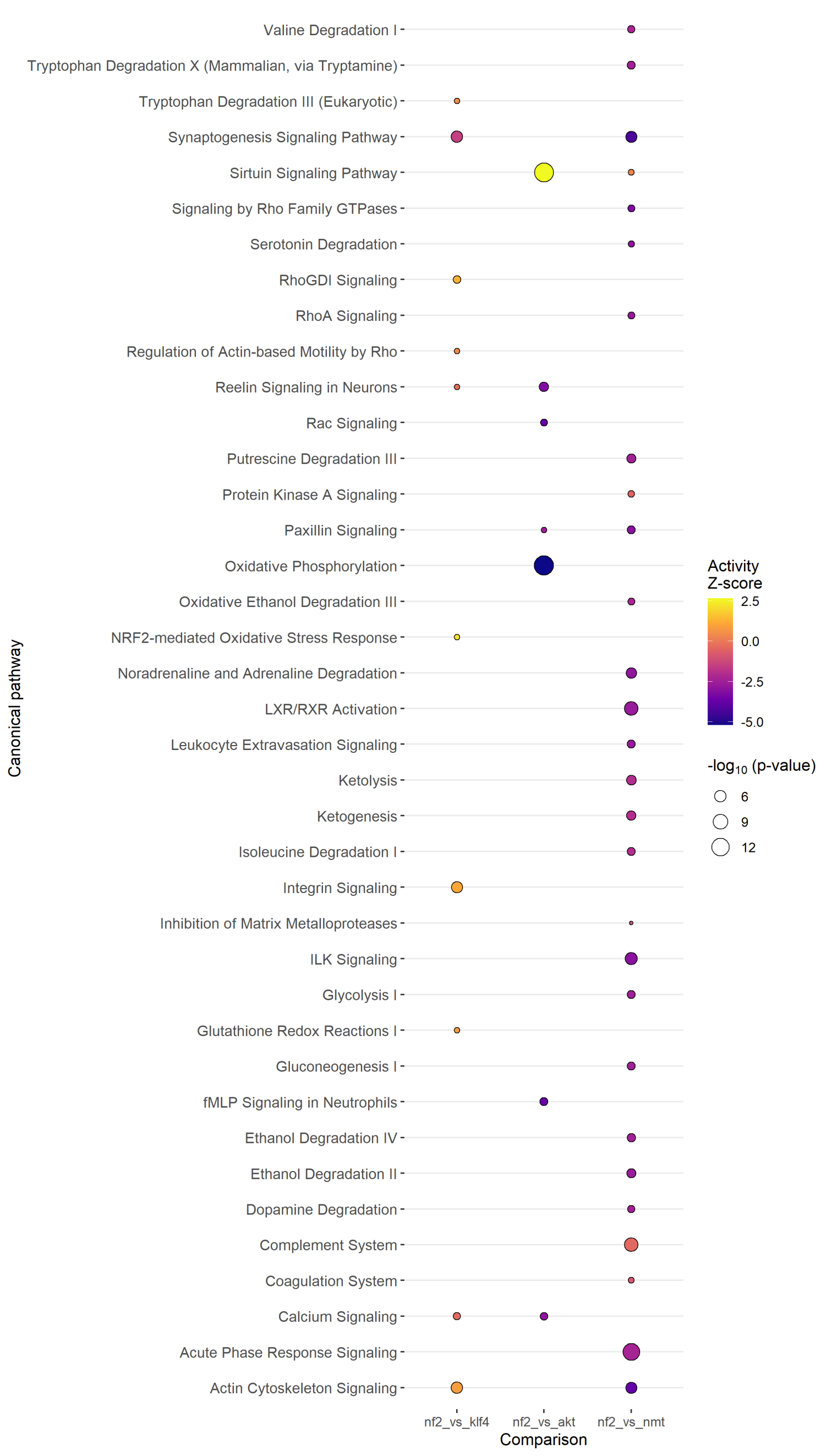

Supplement: Supplementary Fig. S4 — Proteomics IPA analysis NF2−/−. Ingenuity Pathway Analysis (IPA) using proteomic data from tumours holding NF2−/− genomic background showing differential protein expression compared to KLF4K409Q/TRAF7, AKT1E17K/TRAF7, and NMT. Balloon plot shows pathways demonstrating −log10 (p-value) > 3, Upregulated pathways (Z-score ≥ 3) marked by reddish to the yellowish dot; Downregulated pathways (Z-score ≤ −3) marked by purple to the blackish dot. NMT = normal meningeal tissue. [file mmc4.pdf]

**a**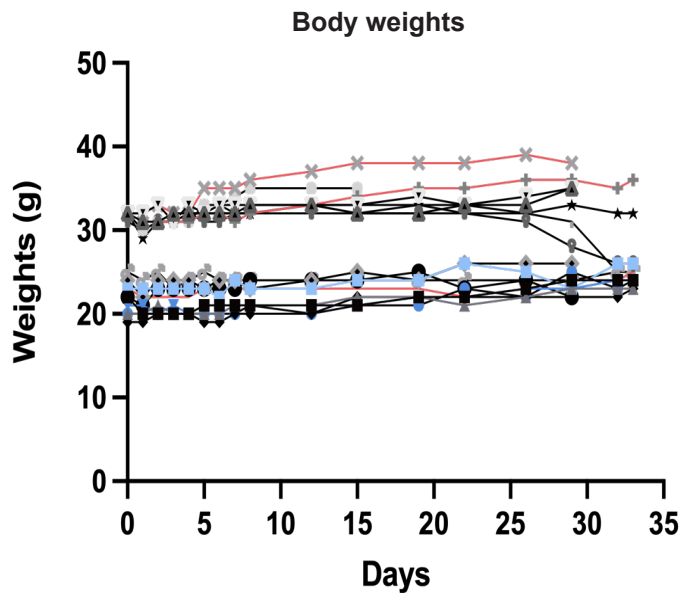**b**

IVIS readings 5 days post-surgery

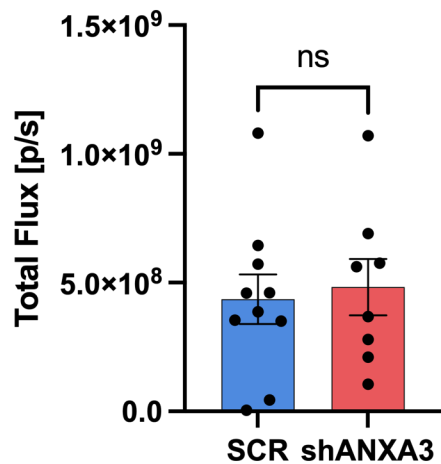**c**

Luminescence (5 days post-surgery)

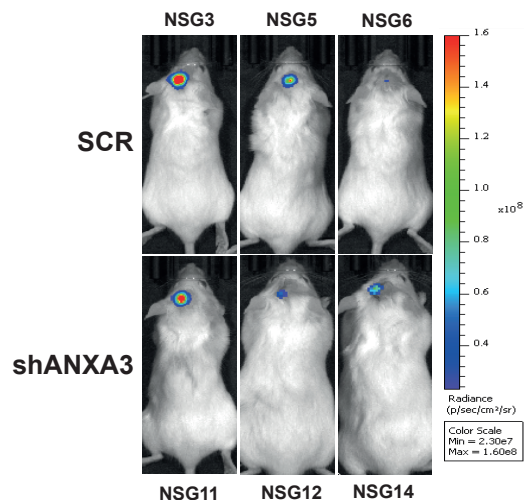

Supplement: Supplementary Fig. S5 — NSG Xenograft Mouse Model Controls. (a) Animal weights (males and females) were monitored from the initial day (pre-surgery) to the final day, with representative animals from Fig. 7 shown in blue (SCR) and red (shANXA3). (b) Tumour growth was quantified through luciferase activity measurements using the IVIS imaging system 5 days post-surgery. (c) Representative animals from Fig. 7 imaged 5 days post-surgery using the IVIS system. A total of 10 animals with SCR cells and 8 animals with shANXA3 cells were used in 2 independent experiments. Scale bars show maximum and minimum luminescence signals; all images were set to the same scale. Statistical significance was assessed using unpaired two-tailed t-tests for two-sample comparisons assuming normal distribution. Significance levels will be indicated by ∗ < 0.05, ∗∗ < 0.01, and ∗∗∗ < 0.001. [file mmc5.pdf]

**a**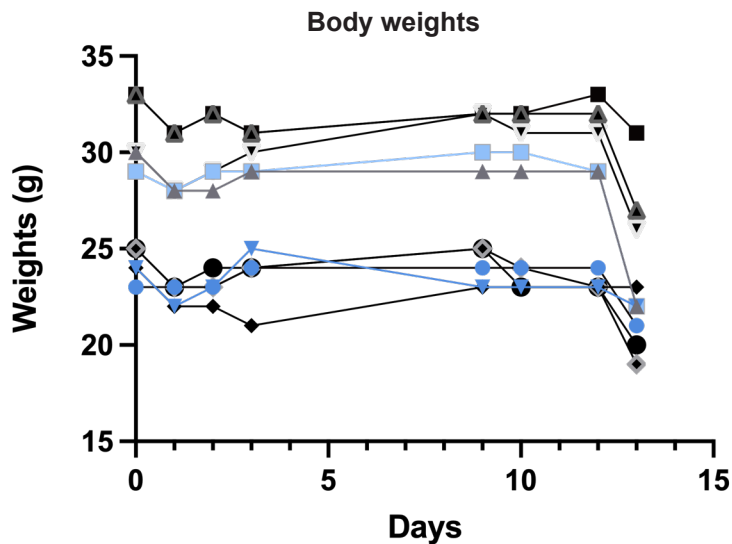**b**

IVIS readings 3 days post-surgery

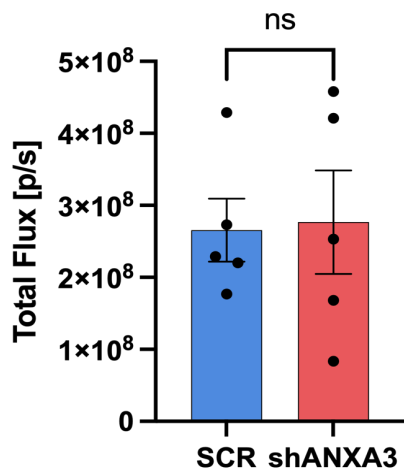**c**

Luminescence (3 days post-surgery)

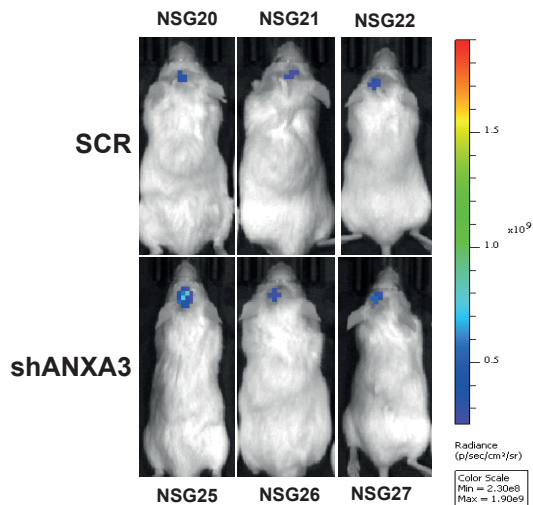

Supplement: Supplementary Fig. S7 — NSG Xenograft Mouse Model Controls. (a) Animal weights (males and females) were monitored from the initial day (pre-surgery) to the final day, with representative animals from Supplementary Fig. S6. (b) Tumour growth was quantified through luciferase activity measurements using the IVIS imaging system 3 days post-surgery. (c) Representative animals from Supplementary Fig. S6 imaged 3 days post-surgery using the IVIS system. Scale bars show maximum and minimum luminescence signals; all images were set to the same scale. Statistical significance was assessed using unpaired two-tailed t-tests for two-sample comparisons assuming normal distribution. Significance levels will be indicated by ∗ < 0.05, ∗∗ < 0.01, and ∗∗∗ < 0.001. [file mmc7.pdf]

# ANXA3

\*\*\*

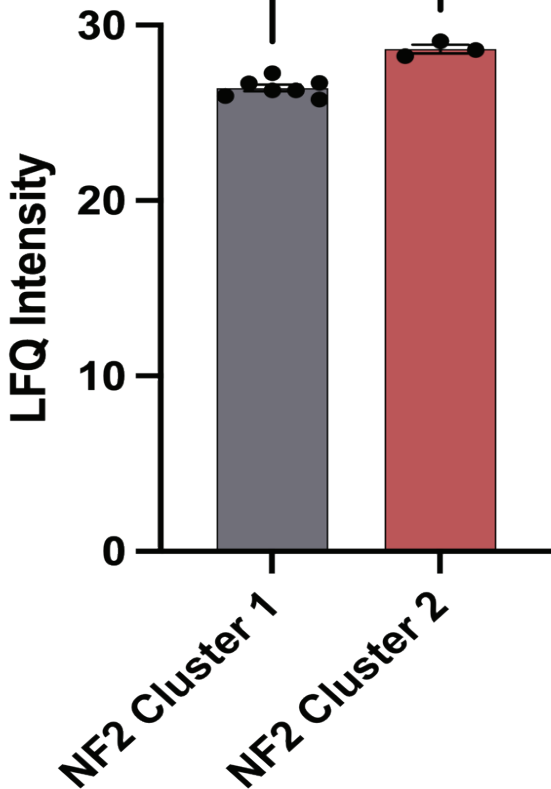

Supplement: Supplementary Fig. S8 — Expression ANXA3 in the NF2 Clusters. Mass spectrometry data analysis shown in Fig. 3 was used to quantify ANXA3 levels in NF2 Cluster 1 (7 samples) and NF2 Cluster 2 (3 samples). Statistical comparisons between the two clusters were performed using unpaired two-tailed t-tests, assuming a normal distribution. Statistical significance is indicated as follows: ∗p < 0.05, ∗∗p < 0.01, and ∗∗∗p < 0.001. [file mmc8.pdf]
