# Supplementary material for: Integrated proteomic and targeted Next Generation Sequencing reveal relevant heterogeneity in lower-grade meningioma and ANXA3 as a new target in NF2 mutated meningiomas
Source: eBioMedicine. 2025 Jun 24;117:105814. doi: 10.1016/j.ebiom.2025.105814 (PMC12278414; doi:10.1016/j.ebiom.2025.105814)
Supplement: Supplementary Fig. S6 — CH157MN GFP-LUC2 NF2−/− ANXA3 KD in vitro shows reduced proliferation and in vivo loses ANXA3 KD. (a) ANXA3 KD in CH157MN GFP-LUC2 NF2−/− grade 3 immortalised cells over-expressing GFP with the quantification of ANXA3 and the evaluation of the proliferation marker MCM2 normalised by GAPDH. (b) EdU proliferation assay showing the microscopy and % of EdU-positive cells. Experiments were carried out in three independent experiments. One-Way ANOVA with Dunnett’s multiple comparisons test with simple pooled variance was employed for experiments with more than two samples assuming normal distribution, alongside non-parametric tests (Friedman's test) if data is not normal. (c) Representative bioluminescence images of NSG mice engrafted with SCR (top panel) and ANXA3 KD (bottom panel) CH157MN GFP-LUC2 cells, 13 days post-surgery, with luciferase signal directly correlating to tumour size. (d) Representative fluorescent images overlaid on the skull convexity, showing the ex vivo GFP signal from tumour cells (days post-surgery indicated in the bottom right panel). (e) Quantification of tumour growth represented as fold-increase in bioluminescence normalised to 3d post-surgery and Western blots using samples from the same animals described in (d) showing the loss of ANXA3 KD in vivo. Total of 5 animals with SCR cells and 5 animals with shANXA3 cells were used in 1 independent experiment and the cell growth was monitored up to 13 days post-surgery and Representative images of 3 animals showing SCR and shANXA3, respectively. Scale bars show maximum and minimum luminescence and GFP signals, all images were set to the same scale. Statistical significance was assessed using unpaired two-tailed t-tests for two-sample comparisons assuming normal distribution. Significance levels will be indicated by ∗ < 0.05, ∗∗ < 0.01, and ∗∗∗ < 0.001. Annexin expression was measured as a double band at 36 and 33 KDa. [file mmc6.pdf]

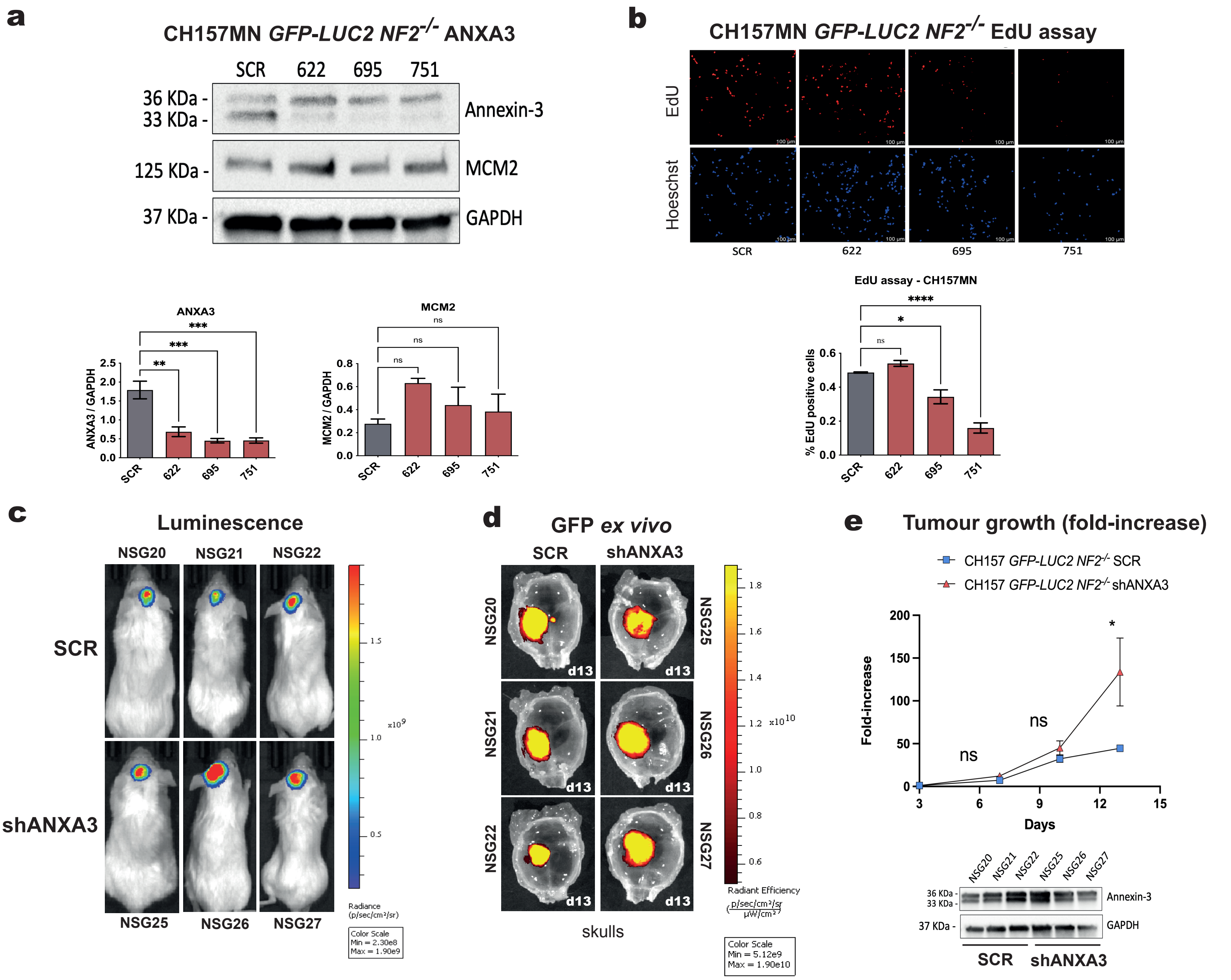
a
CH157MN GFP-LUC2 NF2-/- ANXA3
SCR
622
695
751
36 KDa -
33 KDa -
Annexin-3
125 KDa -
MCM2
37 KDa -
GAPDH
ANXA3
ANXA3 / GAPDH
MCM2
MCM2 / GAPDH
ns
b
CH157MN GFP-LUC2 NF2-/- EdU assay
EdU
Hoeschst
EdU assay - CH157MN
% EdU positive cells
c
Luminescence
NSG20
NSG21
NSG22
SCR
shANXA3
NSG25
NSG26
NSG27
Radiance (p/sec/cm²/sr)
Color Scale Min = 2.30e8 Max = 1.90e9
d
GFP ex vivo
d13
skulls
Radiant Efficiency (p/sec/cm²/sr / μW/cm²)
Color Scale Min = 5.12e9 Max = 1.90e10
e
Tumour growth (fold-increase)
CH157 GFP-LUC2 NF2-/- SCR
CH157 GFP-LUC2 NF2-/- shANXA3
Fold-increase
Days
Annexin-3
GAPDH
