# Supplementary material for: Integrated proteomic and targeted Next Generation Sequencing reveal relevant heterogeneity in lower-grade meningioma and ANXA3 as a new target in NF2 mutated meningiomas
Source: eBioMedicine. 2025 Jun 24;117:105814. doi: 10.1016/j.ebiom.2025.105814 (PMC12278414; doi:10.1016/j.ebiom.2025.105814)
Supplement: Supplementary Western blots [file mmc13.pdf]

# *AKT1 $^{E17K}$/TRAF7* vs all

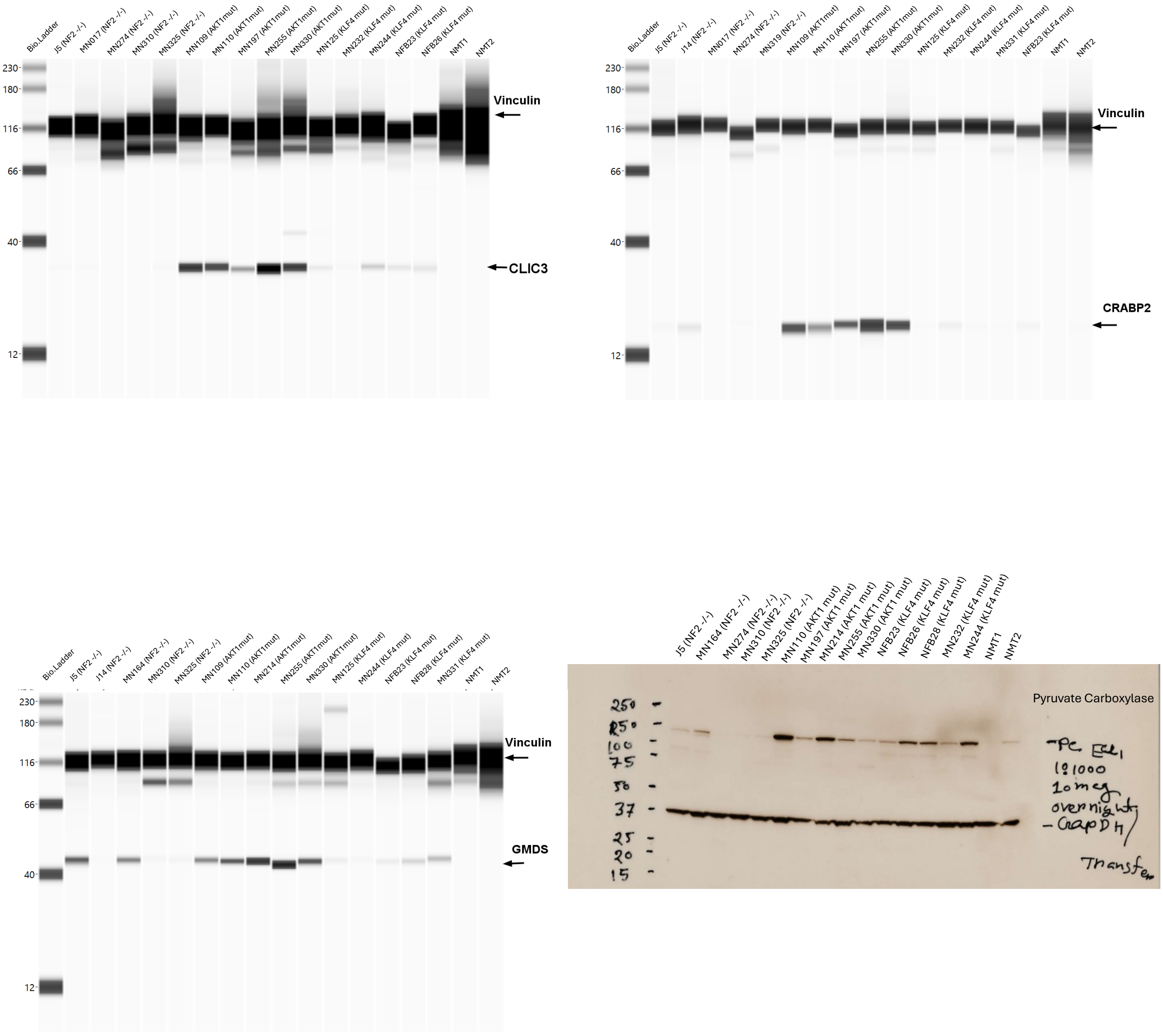


**Figure 4a**

## ***KLF4 $^{K409Q}$/TRAF7* vs all**

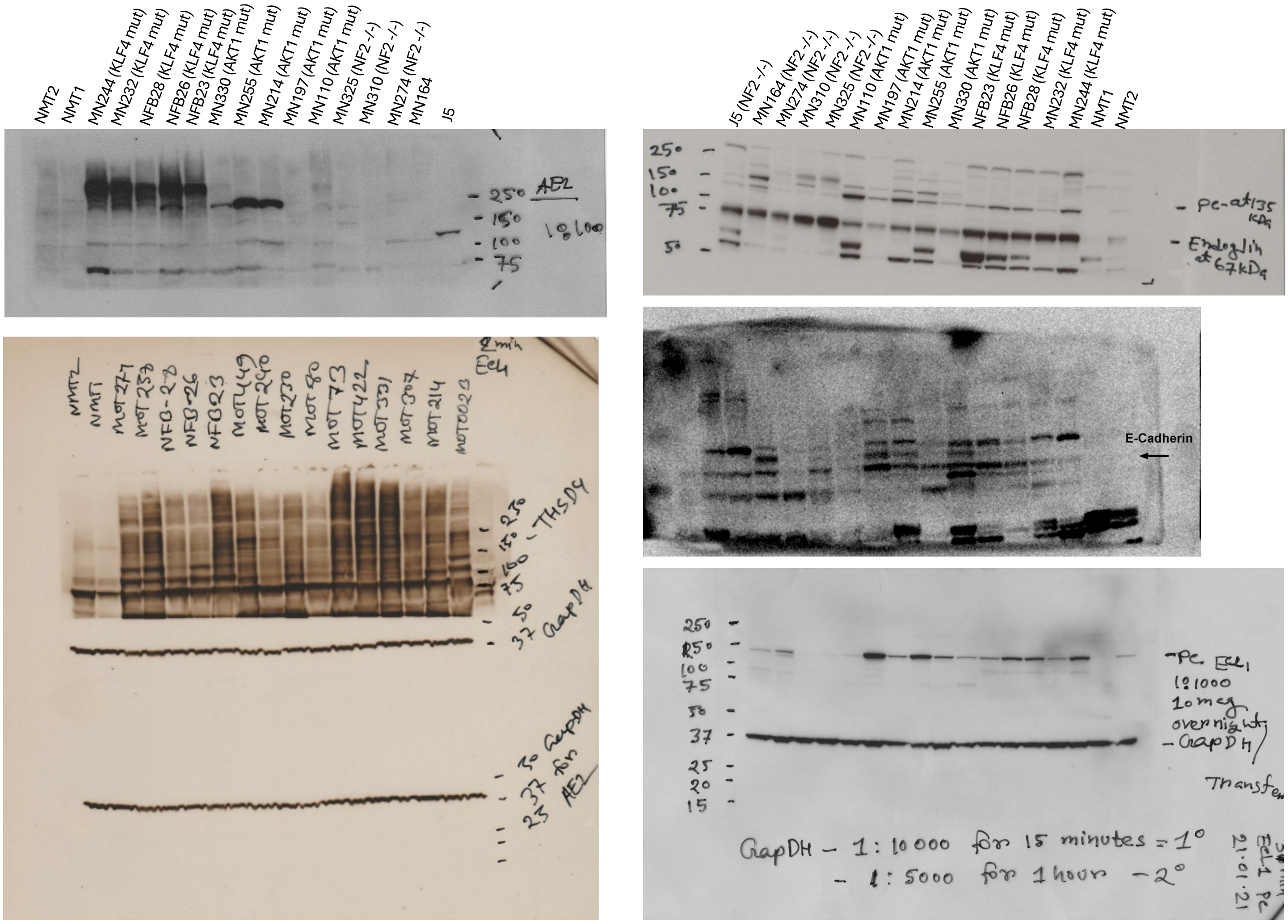


**Figure 4b**

## ***NF2*** $^{-/-}$ **vs all**

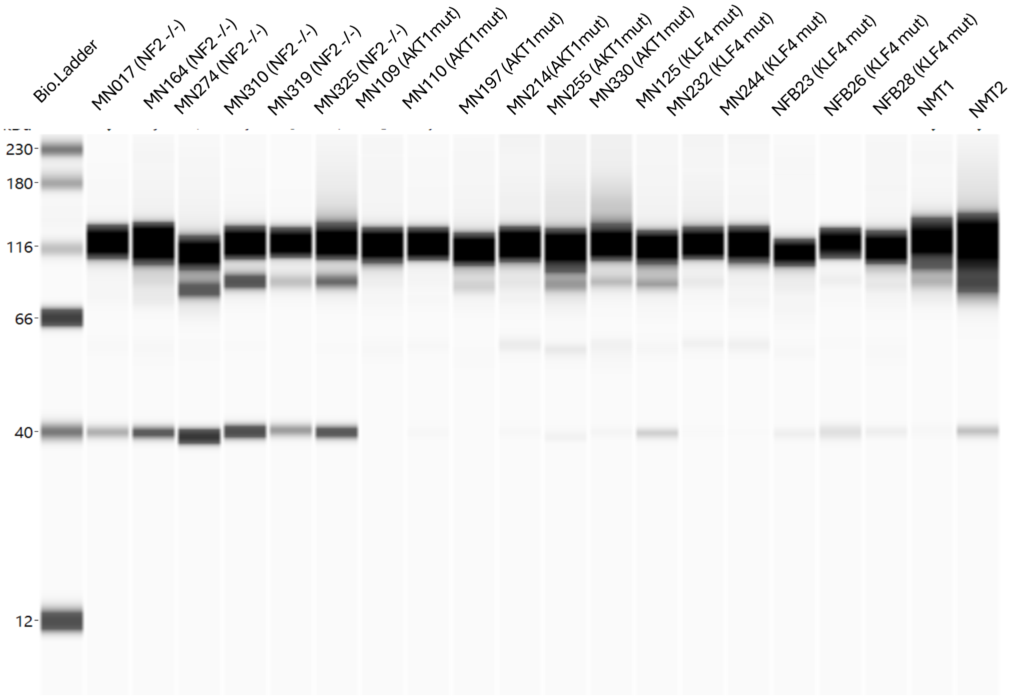


Annexin-3

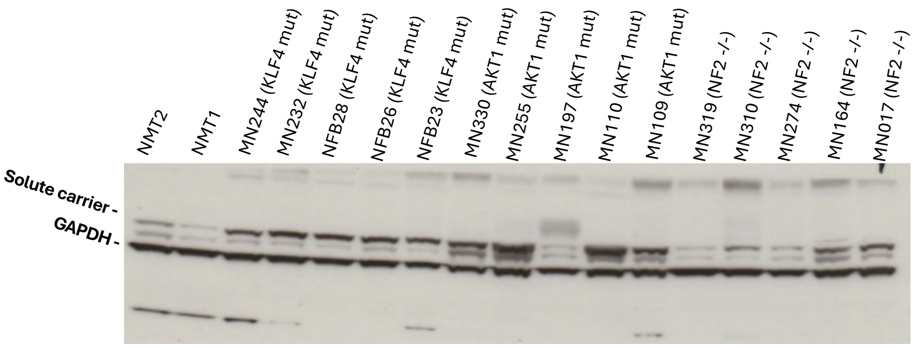


**Figure 4c**

## ANXA3 expression in ANXA3 KD primary cells

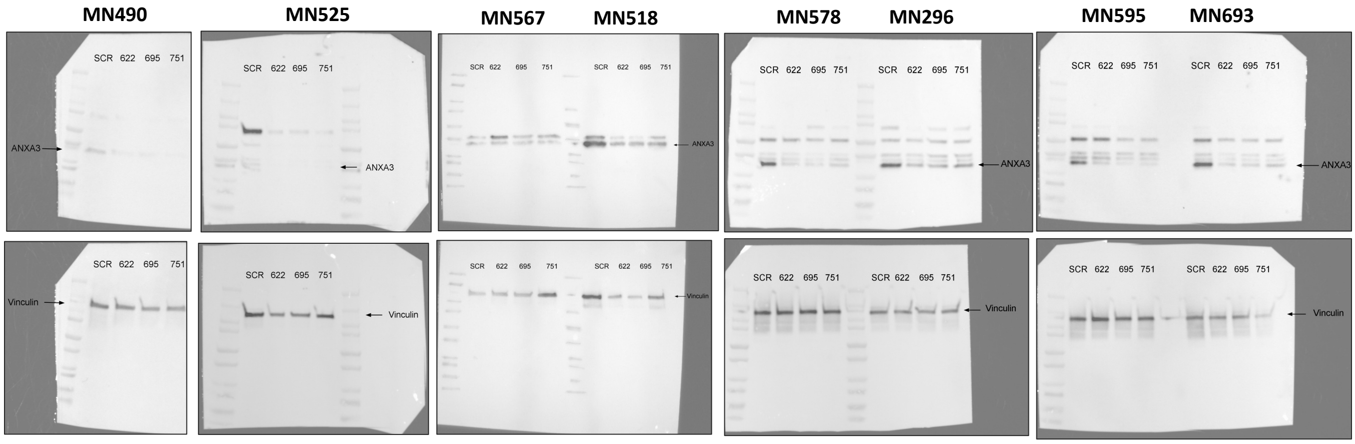


## MCM2 expression in ANXA3 KD primary cells

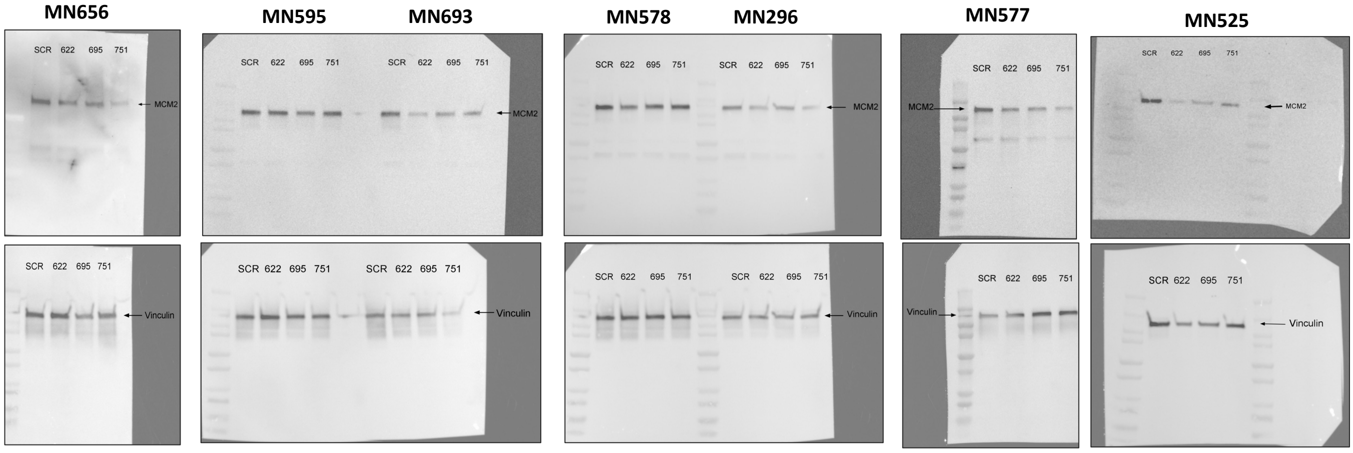


## Total ERK and pERK expression in ANXA3 KD primary cells

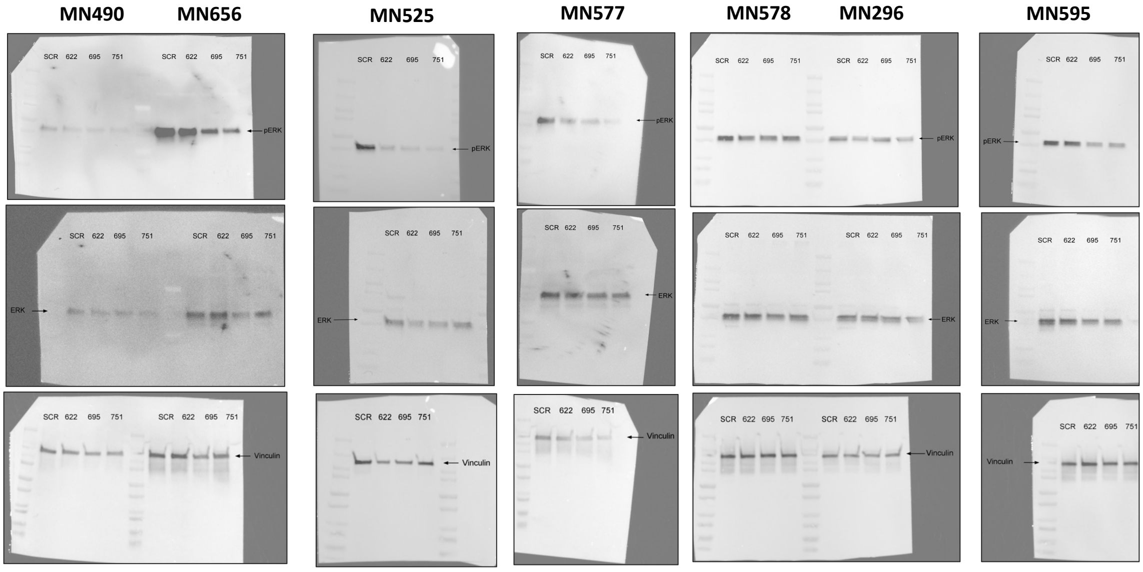


**Figure 6e & d**

# ANXA3 expression in ANXA3 KD Ben-Men-1

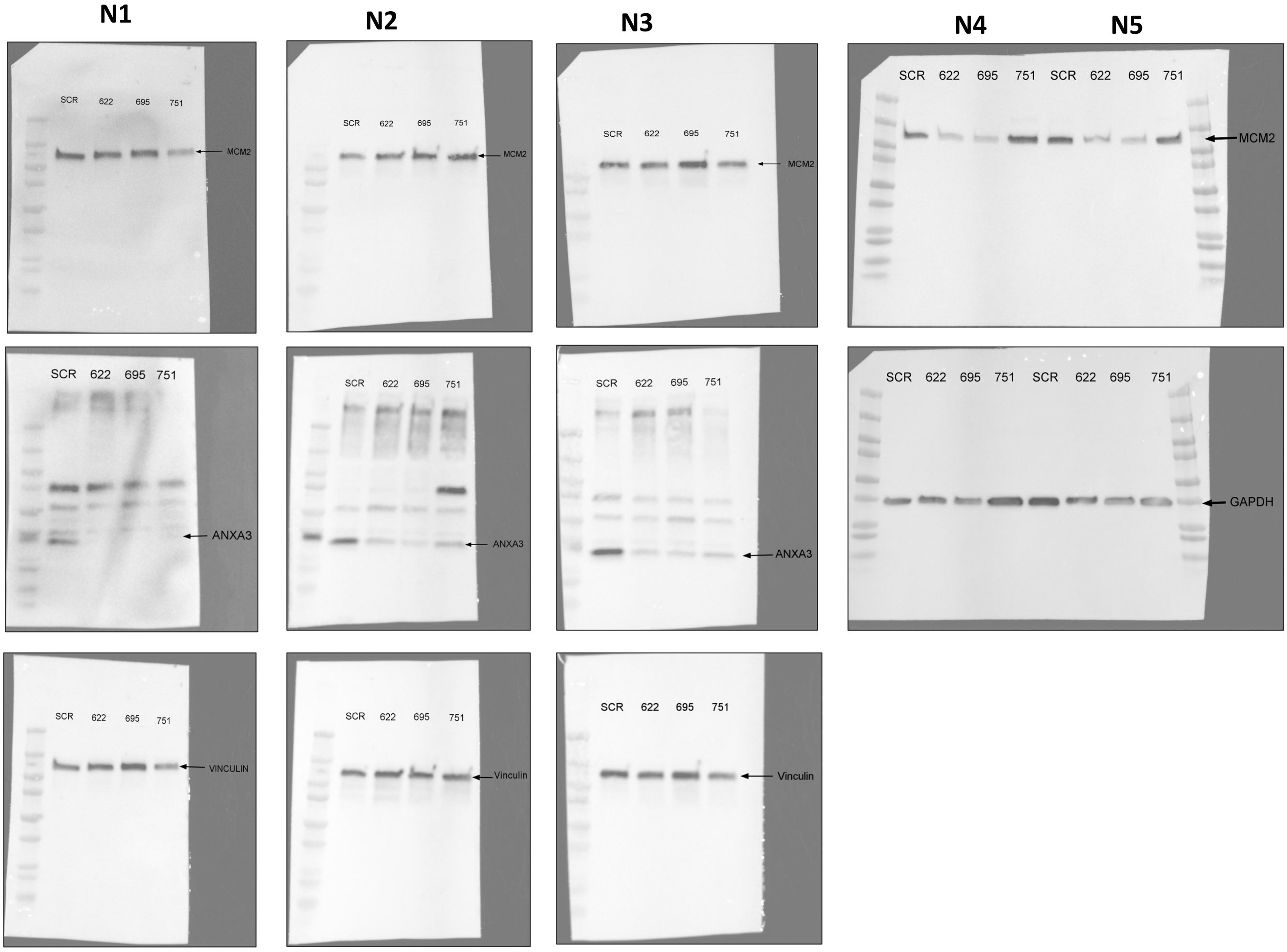


# Figure 6a

# ANXA3 expression in NF2 null meningioma tumours

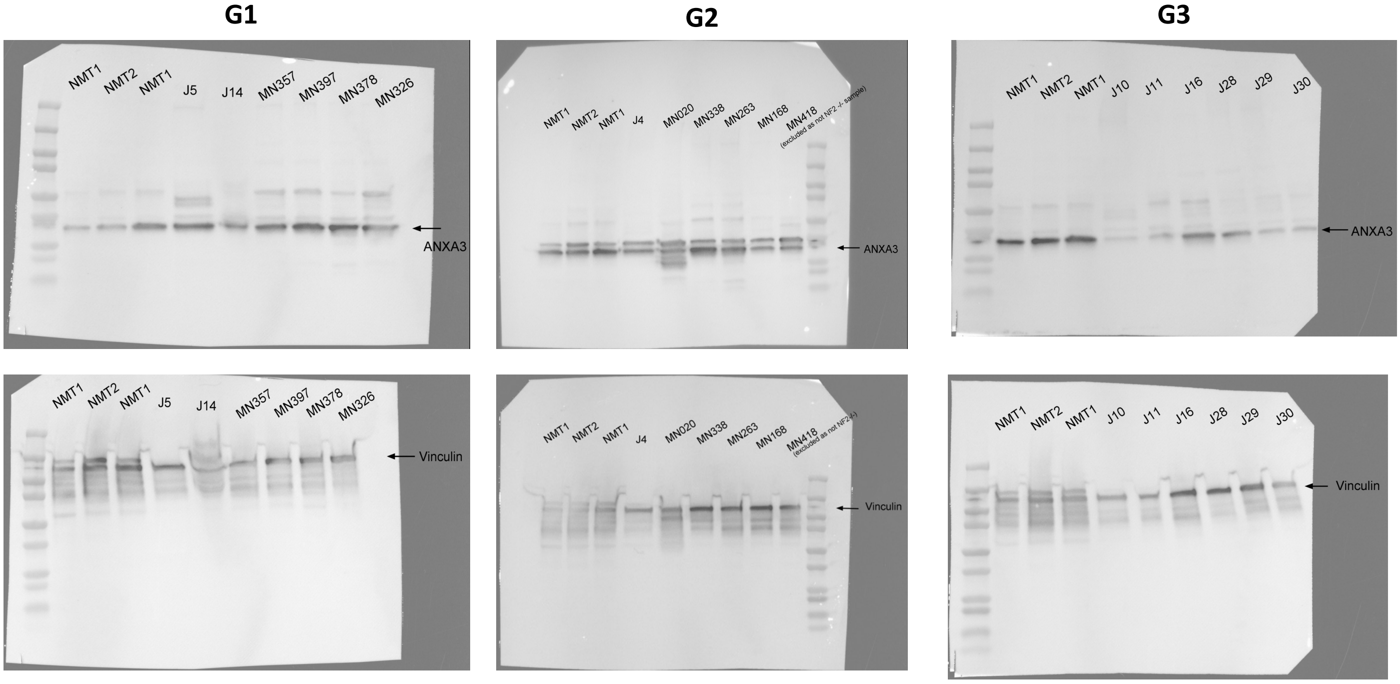


Figure 7a

# ANXA3 expression in ANXA3 KD NCH93

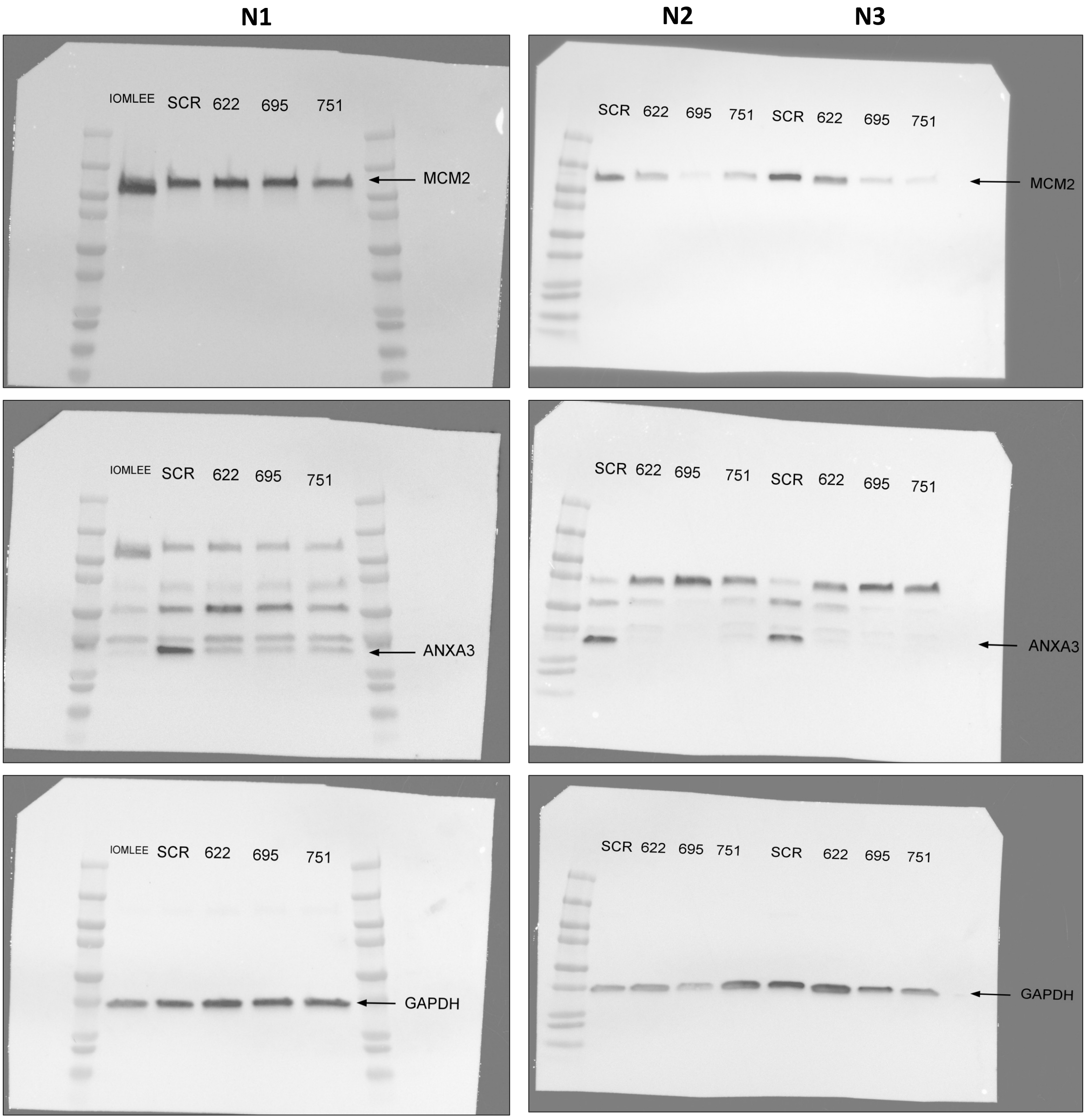


Figure 7b

# ANXA3 expression in ANXA3 KD CH157invitro

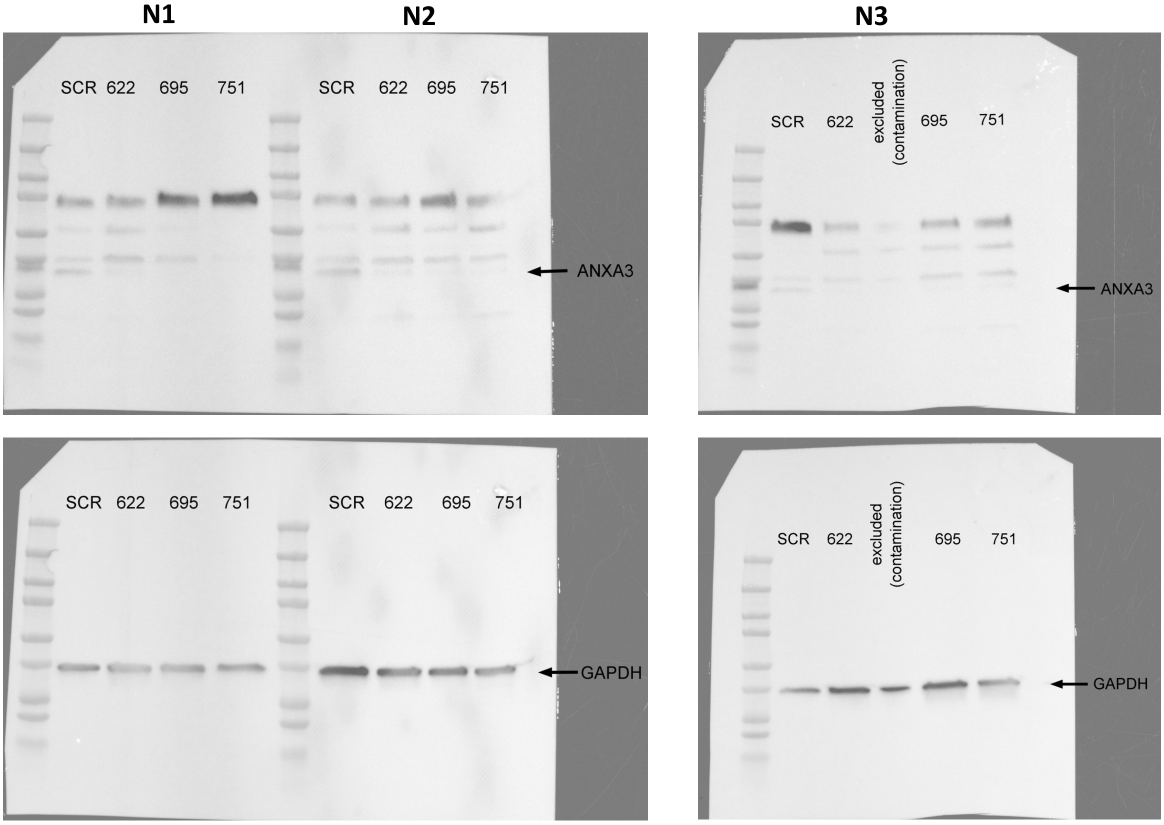


Supplementary figure S6a

# ANXA3 expression in ANXA3 KD CH157invitro

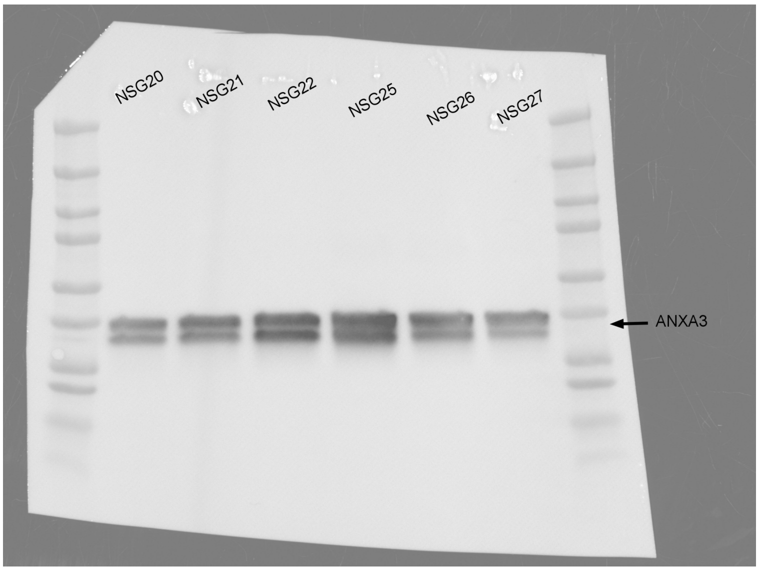


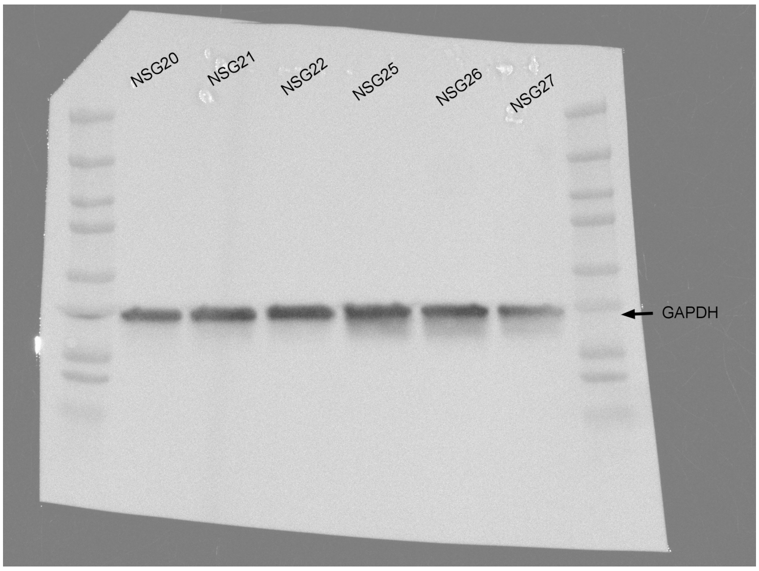


# Supplementary figure S6a
