# Supplementary material for: Integrated proteomic and targeted Next Generation Sequencing reveal relevant heterogeneity in lower-grade meningioma and ANXA3 as a new target in NF2 mutated meningiomas
Source: eBioMedicine. 2025 Jun 24;117:105814. doi: 10.1016/j.ebiom.2025.105814 (PMC12278414; doi:10.1016/j.ebiom.2025.105814)
Supplement: Reagent validation file [file mmc14.docx]

| **Supplier** | **Antibody** | **Cat.n.** | **Source/**  **Isotype** | **Clone** | **Dilution used** | **Method validation (supplier)** |
| --- | --- | --- | --- | --- | --- | --- |
| Sigma | Anti-ANXA3 | HPA013398 | Rabbit IgG | Polyclonal | 1/1000 | IHC, WB, ICC |
| Sigma | Anti-Vinculin | V9131 | Mouse IgG1 | Monoclonal | 1/800 | ICC, IHC, WB |
| Abcam | Anti-GAPDH | ab8245 | mouse IgG1 | Monoclonal | 1/10000 | ICC, ICC, WB |
| Cell Signaling Technology | Anti-Phospho-p44/42 MAPK (Erk1/2) (Thr202/Tyr204) | 9101 | Rabbit | Polyclonal | 1/1000 | ICC, WB, IP |
| Cell Signaling Technology | Anti-p44/42 MAPK (Erk1/2)(137F5) | 4695S | Rabbit IgG | Monoclonal | 1/1000 | ICC, WB, IP, ICC, IHC |
| Cell Signaling Technology | Anti-MCM2 (D7G11) XP | 3619 | Rabbit IgG | Monoclonal | 1/1000 | IF, WB, IP, IHC, ChIP |
| Sigma | Anti-CLIC3 | HPA005963 | Rabbit IgG | Polyclonal | 1/75 | ICC, IHC |
| Sigma | Anti-CRABP2 | HPA004135 | Rabbit IgG | Polyclonal | 1/50 | ICC, IHC |
| Novus | Anti-GMDS | NBP1-33424 | Rabbit IgG | Polyclonal | 1/50 | WB, IHC |
| Sigma | Anti-Pyruvate Carboxylase | HPA043922 | Rabbit IgG | Polyclonal | 1/500 | ICC, IHC |
| Sigma | Anti-Endoglin | HPA011862 | Rabbit IgG | Polyclonal | 1/500 | IHC |
| Sigma | Anti-E-Cadherin | SAB4503751 | Rabbit IgG | Polyclonal | 1/500 | ICC, IHC, WB |
| Sigma | Anti-APEP2 | SAB2701184 | Rabbit | Polyclonal | 1/500 | IHC, ICC |
| Novus | Anti-Solute Carrier Family 29 member 1 | NBP3-02977 | Rabbit IgG | Polyclonal | 1/500 | WB |
| Thermo Fisher | Anti-Mouse | 1706516 | Goat IgG | Polyclonal | 1/5000 | WB, IHC, ELISA, IP |
| Thermo Fisher | Anti-Rabbit | 1706515 | Goat IgG | Polyclonal | 1/5000 | WB, IHC, ELISA |

*Abbreviations: WB=Western blot, IHC=Immunohistochemistry, ICC=Immunocytochemistry, IP=Immunoprecipitation, ChIP=Chromatin immunoprecipitation, ELISA=Enzyme-Linked immunosorbent assay.

All antibodies used in this study were commercially sourced and validated by the supplier, who also provides references to publications from various institutions that have used the same antibodies.
